# Large-Scale Mapping and Validation of Escherichia coli Transcriptional Regulation from a Compendium of Expression Profiles

**DOI:** 10.1371/journal.pbio.0050008

**Published:** 2007-01-09

**Authors:** Jeremiah J Faith, Boris Hayete, Joshua T Thaden, Ilaria Mogno, Jamey Wierzbowski, Guillaume Cottarel, Simon Kasif, James J Collins, Timothy S Gardner

**Affiliations:** 1 Bioinformatics Program, Boston University, Boston, Massachusetts, United States of America; 2 Department of Biomedical Engineering, Boston University, Boston, Massachusetts, United States of America; 3 Boston University School of Medicine, Boston, Massachusetts, United States of America; 4 Department of Computer and Systems Science A. Ruberti, University of Rome, La Sapienza, Rome, Italy; 5 Cellicon Biotechnologies, Boston, Massachusetts, United States of America; Johns Hopkins University, United States of America

## Abstract

Machine learning approaches offer the potential to systematically identify transcriptional regulatory interactions from a compendium of microarray expression profiles. However, experimental validation of the performance of these methods at the genome scale has remained elusive. Here we assess the global performance of four existing classes of inference algorithms using 445 Escherichia coli Affymetrix arrays and 3,216 known E. coli regulatory interactions from RegulonDB. We also developed and applied the context likelihood of relatedness (CLR) algorithm, a novel extension of the relevance networks class of algorithms. CLR demonstrates an average precision gain of 36% relative to the next-best performing algorithm. At a 60% true positive rate, CLR identifies 1,079 regulatory interactions, of which 338 were in the previously known network and 741 were novel predictions. We tested the predicted interactions for three transcription factors with chromatin immunoprecipitation, confirming 21 novel interactions and verifying our RegulonDB-based performance estimates. CLR also identified a regulatory link providing central metabolic control of iron transport, which we confirmed with real-time quantitative PCR. The compendium of expression data compiled in this study, coupled with RegulonDB, provides a valuable model system for further improvement of network inference algorithms using experimental data.

## Author Summary

Organisms can adapt to changing environments—becoming more virulent, for example, or activating stress responses—thanks to a flexible gene expression program controlled by the dynamic interactions of hundreds of transcriptional regulators. To unravel this regulatory complexity, multiple computational algorithms have been developed to analyze gene expression profiles and detect dependencies among genes over different conditions. It has been difficult to judge whether these algorithms can generate accurate global maps of regulatory interactions, however, because of the absence of a model organism with both a compendium of gene expression data and a corresponding network of experimentally determined regulatory interactions. To address this issue, we assembled 445 Escherichia coli microarrays, applied four classes of inference algorithms to the dataset, and validated the predictions against 3,216 experimentally determined E. coli interactions. The top-performing algorithm identifies 1,079 regulatory interactions at a confidence level of 60% or higher. Of these predicted interactions, 741 are novel and illuminate the regulation of amino acid biosynthesis, flagella biosynthesis, osmotic stress response, antibiotic resistance, and iron regulation. By defining the capabilities and limitations of network inference algorithms for large-scale mapping of prokaryotic regulatory networks, our work should facilitate their application to the mapping of novel microbes.

## Abstract 3

A novel, machine-learning method is developed to predict transcriptional regulatory interactions, making use of microarray data. One interaction identified appears to be important for the control of iron transport.

## Introduction

High-throughput genome sequencing and bioinformatics technologies have dramatically eased the task of genomic annotation, producing parts lists of living organisms as simple as Mycoplasmas and as complex as mammals. Further progress in the understanding of an organism's biology requires development and refinement of techniques to determine the dynamic interactions among an organism's molecular parts [1]. A major difficulty of this task is the context-specific nature of gene regulation. The total space of possible transcriptional regulatory interactions for an organism is the number of transcription factors multiplied by the number of genes multiplied by the number of environmental contexts in which the cell might find itself. Methods to identify regulatory interactions must efficiently determine the thousands of true regulatory interactions out of the billions of possible ones.

Pioneering efforts to identify regulatory interactions on a genome scale have used machine-learning algorithms to identify *cis*-regulatory motifs or transcription factor target genes using a large set of expression arrays [2–18], genome-wide location analysis chromatin immunoprecipitation (ChIP-Chip) [19,20], or a combination of these and other high-throughput methods [21–26]. In general, the precision of these methods has been evaluated by testing for functional enrichment of co-regulated genes, experimental confirmation of a few selected regulatory relationships, or cross-validation within the training dataset. However, experimental validation of the precision of these methods at the genome scale has remained elusive due to the lack of a model organism with both a known regulatory structure and compatible experimental data. Therefore, the relative merits and broader utility of these approaches remain difficult to judge.

Here we demonstrate an unsupervised network inference method, context likelihood of relatedness (CLR), which uses transcriptional profiles of an organism across a diverse set of conditions to systematically determine transcriptional regulatory interactions. We take advantage of the extensive knowledge of transcriptional regulation in Escherichia coli to assess the performance of the CLR algorithm and several other algorithms on a genome scale. In *E. coli,* a set of 3,216 experimentally confirmed regulatory interactions among 1,211 genes have been curated in the RegulonDB database [27], which can be used for performance assessment.

**Figure 1:**
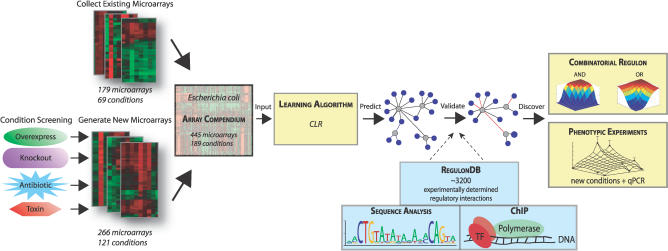
Overview of Our Approach for Mapping the E. coli Transcriptional Regulatory Network Microarray expression profiles were obtained from several investigators. Our laboratory profiled additional conditions, focusing on DNA damage, stress responses, and persistence. These two data sources were combined into one uniformly normalized E. coli microarray compendium that was analyzed with the CLR network inference algorithm. The predicted regulatory network was validated using RegulonDB, sequence analysis, and ChIP. The validated network was then examined for cases of combinatorial regulation, one of which was explored with follow-up real-time quantitative PCR experiments.

We assembled a compendium of 445 new and previously published E. coli Affymetrix Antisense2 microarray expression profiles collected under various conditions including pH changes, growth phases, antibiotics, heat shock, different media, varying oxygen concentrations, and numerous genetic perturbations (Figure 1 and Table 1). This compendium, combined with the knowledge in RegulonDB, allowed us to assess the genome-scale performance of the CLR algorithm and multiple other unsupervised network inference algorithms applied to experimental data. The compendium and algorithms are available on the Many Microbe Microarrays database (M^3D^) Web site (http://m3d.bu.edu/). We included several versions of relevance networks (the foundation for the CLR algorithm) [28,29], ARACNe [30], Bayesian networks [7], and regression networks in this algorithm comparison. CLR, the top-performing inference method, predicted 1,079 regulatory interactions at a 60% true positive rate. Sequence analysis of the promoters of the inferred gene targets yielded many known and novel promoter motifs. We also tested, via ChIP, more than 250 of the interactions inferred for three transcription factors at all confidence levels to verify the algorithm precision estimates based on RegulonDB and to confirm 21 of the novel predictions. In addition, an analysis of the CLR inferred network led to the discovery of an unexpected regulatory link between central metabolism and the regulation of iron import into the cell, which we confirmed with real-time quantitative PCR.

**Table 1:**
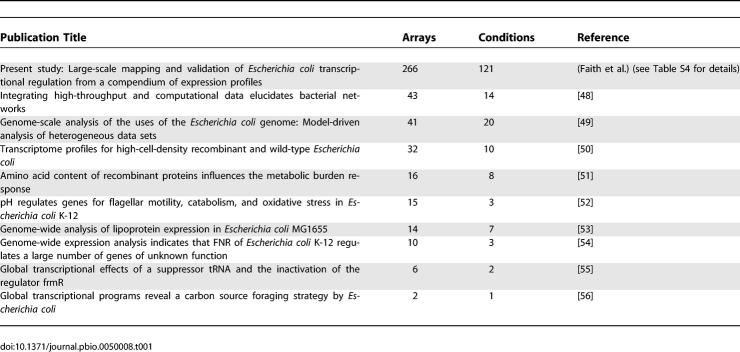
Data Sources for the Escherichia coli Microarray Compendium

## Results/Discussion

### The CLR Algorithm

The CLR algorithm is an extension of the relevance networks approach [28,31] for identifying transcriptional regulatory interactions. Although the relevance networks algorithm was written primarily for clustering, its authors also suggest its utility for identifying regulatory networks [29]. The original relevance networks method used mutual information for scoring the similarity between the expression levels of two genes in a set of microarrays. A gene and a transcription factor are predicted to interact if the mutual information between the expression levels of the gene and its potential regulator is above some set threshold. Like correlation, mutual information is a metric that detects statistical dependence between two variables. But unlike correlation, it does not assume linearity, continuity, or other specific properties of the dependence [32,33]. As such, mutual information possesses the flexibility to detect regulatory interactions that might be missed by linear measures such as the correlation coefficient.

In the relevance networks algorithm, there are tradeoffs between true positive and false positive rates in choosing a threshold for the identification of significant regulatory interactions. A high threshold results in a smaller network with fewer false positives, but it also eliminates potential novel interactions. Conversely, a low threshold will often capture false positive interactions due to a number of factors, including background correlation and misinterpretation of indirect dependence as direct interaction.

**Figure 2:**
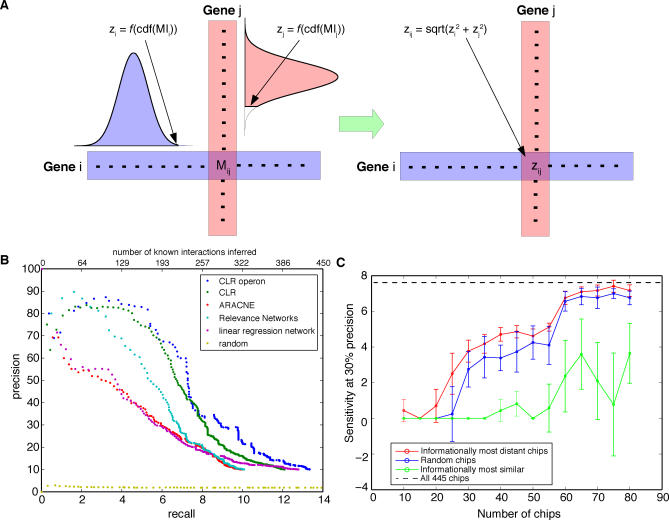
The CLR Algorithm: Methods and Comparison to Other Approaches (A) A schema of the CLR algorithm. The *z*-score of each regulatory interaction depends on the distribution of MI scores for all possible regulators of the target gene *(z_i_)* and on the distribution of MI scores for all possible targets of the regulator gene *(z_j_)*. (B) Precision and recall for several different network inference methods applied to all genes in the E. coli microarray compendium were calculated using RegulonDB. The number of correctly inferred interactions (within RegulonDB) for each recall value is labeled on the top of the chart. All algorithms performed far better than the random method. Both CLR and relevance networks reach high precisions, but CLR attains almost twice the recall of relevance networks at some levels of precision. (C) Using 60 well-chosen arrays, we can infer a network, nearly equivalent in recall and precision to the network inferred using all 445 microarrays in the compendium (dotted horizontal line), reflecting the redundancy of the compendium and the potential for improvement in choosing subsequent perturbations to profile.

The CLR algorithm builds upon the relevance network but applies an adaptive background correction step to eliminate false correlations and indirect influences (Figure 2A). After computing the mutual information between regulators and their potential target genes, CLR calculates the statistical likelihood of each mutual information value within its network context. The algorithm compares the mutual information between a transcription factor/gene pair to the “background” distribution of mutual information scores for all possible transcription factor/gene pairs that include either the transcription factor or its target (Figure 2A). The most probable interactions are those whose mutual information scores stand significantly above the background distribution of mutual information scores. This step removes many of the false correlations in the network by eliminating “promiscuous” cases, where one transcription factor weakly co-varies with a large numbers of genes, or one gene weakly co-varies with many transcription factors. Such promiscuity arises when the assayed conditions are inadequately or unevenly sampled, thus failing to distinguish direct interactions from indirect influences, or when microarray normalization fails to remove false background correlations due to inter-lab variations in methodology.

We applied CLR to the 4,345 genes on the E. coli Antisense2 microarray using the 445 profiles in the compendium to identify the gene targets of the E. coli transcription factors. For comparison, we ran several variants of commonly used network inference algorithms on the compendium data; the top-performing variant of each algorithm is shown in Figure 2 (a detailed comparison of each algorithm and the tested variants is available in Protocol S1). As a point of reference on algorithm performance, we also show the performance attained by randomly guessing interaction scores from a uniform distribution. Interactions for all algorithms were only allowed from 328 known or predicted transcription factors to any of the 4,345 genes, enabling clear biological interpretation, assignment of direction (from transcription factors to non–transcription factor genes), and validation of the predictions. Interactions were also identified between transcription factors, but direction was not assigned.

To score our results, we compared our predicted interactions with the set of 3,216 interactions in the RegulonDB database [27]. We computed two measures: recall, which is the fraction of the 3,216 known E. coli interactions that CLR successfully identified; and precision, which is the fraction of identified interactions that are true positives (Figure 2B). RegulonDB contains regulatory information for about one-fourth of the genes in the E. coli genome and about one-half of the transcription factors. Thus, it is large enough to make sound estimates of algorithm performance at the genome scale. Yet, we caution that the accuracy of our performance estimates may be biased by the incomplete nature of this dataset. We also verified the accuracy of these performance estimates by performing ChIP experiments on a large number of interactions, inferred by CLR, that are not present in RegulonDB.

**Figure 3:**
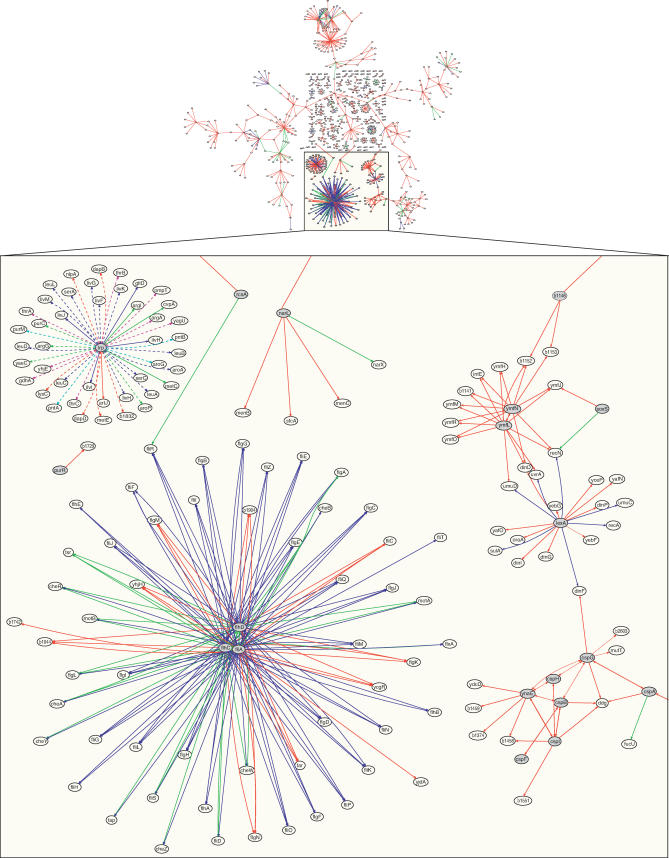
The Transcriptional Regulatory Map Inferred by CLR with an Estimated 60% Precision The precision of the network is obtained by measuring the percentage of correctly inferred edges (blue lines) out of all the predicted edges for genes with known connectivity (blue lines and green lines). The green edges represent false positives based on RegulonDB. The red edges connect genes/regulators not present in RegulonDB. A portion of the regulatory map containing many of the Lrp interactions is shown in the expanded box. Dotted lines were tested by ChIP. Magenta and cyan dotted lines are previously unknown targets of Lrp, experimentally verified by ChIP. Genes attached to cyan lines previously had no known regulator, whereas magenta indicates a gene that had at least one previously known regulator.

**Figure 4:**
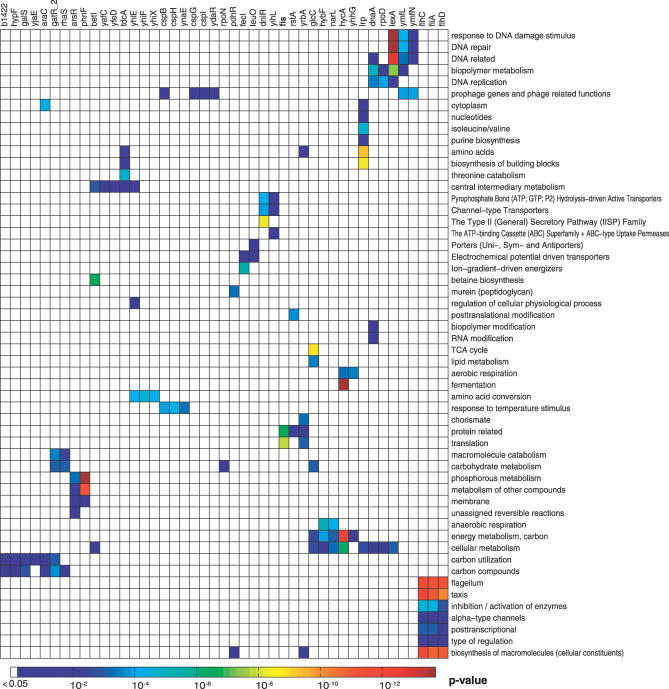
Annotation of Transcription Factor Function by Functional Enrichment Using Predicted Targets from the 60% Precise Network The functional categories of the target genes of each transcription factor were tested for enrichment by a hypergeometric test. Enriched functions indicate which aspects of cellular physiology were most represented in the inferred regulatory interactions. These enriched categories also reflect the conditions sampled in the microarray compendium.

CLR outperforms all other algorithms run on the compendium (Figure 2B). With 60% precision (CLR threshold *z*-score = 5.78), CLR recovers a total of 1,079 regulatory interactions—338 of these among genes included in RegulonDB (Figure 3, blue and green edges) and 741 novel interactions not present in RegulonDB (Figure 3, red edges). In addition, the targets of many transcription factors in this network are significantly enriched for one or more biological functions (Figure 4), and the enriched biological functions reflect the conditions sampled in the microarray compendium. CLR also scores 426 of the 1,079 interactions at a higher confidence of 80%. All 426 interactions identified at 80% precision (CLR threshold *z*-score = 6.92) are illustrated in Figure S1. All identified interactions are available on the M^3D^ Web site (http://m3d.bu.edu) as a graphical map and as tab-delimited text files.

Although we identified hundreds of known regulatory interactions correctly at high precision, this represents only a fraction of known interactions in E. coli. The recall of the algorithm depends on several factors, including the number and diversity of expression profiles. As discussed in Protocol S1, the CLR algorithm can achieve maximum recall and precision using as few as 60 expression profiles selected for maximum diversity (Figure 2C). Large environmental perturbations are the most common conditions amongst these 60 profiles, suggesting environmental perturbations are generally more informative than genetic perturbations for network inference, in agreement with earlier studies (see Protocol S1 for details) [2]. The remainder of the profiles in the compendium contribute mainly redundant information about gene expression responses and regulatory interactions. Thus, the recall achieved by the CLR algorithm appears to be limited largely by the low phenotypic diversity of the dataset. This conclusion is supported by a more detailed analysis of the recovered interactions. For transcription factors with at least two predicted targets, the mean recall per transcription factor is 47% (Figure S2), supporting the idea that when a transcription factor and its targets are adequately perturbed in the dataset, high recall is obtained. For example, when minimal media conditions are included in the compendium, nearly all targets of Lrp are identified (Lrp is a regulator of multiple biosynthetic operons). But when those conditions are removed, the algorithm fails to identify any Lrp targets (see Protocol S1 for details).

### Discovery of Novel Regulatory Pathways

We used the regulatory network predicted by CLR to explore and validate newly inferred regulatory interactions. We confirmed the algorithm's performance using two additional approaches (Figure 1). First, we applied the tools of sequence analysis to discover new regulatory motifs in the promoters of the regulated genes. Second, we performed ChIP experiments to verify many of the novel interactions identified by the algorithm.

#### Further validation of CLR by sequence analysis of regulatory motifs.

Using the set of gene targets predicted for each transcription factor, we applied sequence analysis algorithms to infer the sequence motif bound by each regulator. Not all transcription factors have enough targets to allow reliable motif detection, but for those that do, the motif provides a specific location for the regulatory interaction. A significant sequence motif for a group of genes provides an additional level of validation, as it is unlikely that the group of genes would share a common motif but not a common transcription factor regulator. To detect sequence motifs, we selected all transcription factors predicted to regulate five or more operons with at least a 60% confidence (61 total). For each group of operons regulated by the same transcription factor, we analyzed approximately 150 base pairs upstream of the transcription start site with the MEME multiple alignment system [34].

LexA, a major regulator of DNA repair, is known to have a single well-conserved DNA-binding motif. It is one of the best-perturbed regulators in the microarray compendium due to the compendium's emphasis on DNA-damaging conditions. Consequently, the LexA protein has a large set of correctly predicted targets and exhibits a highly significant motif almost identical to the known canonical LexA motif (Figure S3A). Five out of eight promoters containing the LexA motif in Figure S3A are known LexA targets according to RegulonDB. The other three promoters for *dinI, dinP,* and *yebG* are confirmed LexA targets [35] but are not catalogued in RegulonDB.

Figures S3C and S3D illustrate this approach applied to two putative regulators, YmfN and YnaE. YmfN is a putative DNA-binding protein homologous to a phage terminase. We found a strong motif (*p* value ≈ 0.0061) in all six of the operons inferred for this transcription factor by CLR (Figure S3D). The gene *ymfN* attains its highest levels of expression in our compendium upon exposure to norfloxacin, a DNA-damaging bactericidal agent, and its inferred targets show enrichment in prophage and DNA repair categories (Table S1).

YnaE (Rac prophage) is another putative DNA-binding protein. The latest computational annotation for YnaE available in EcoCyc (http://ecocyc.org/) suggests that its function is also phage-related [36]. There is enrichment for cold-shock response proteins in the predicted YnaE regulon (Table S2). Also present are rhsE, a stationary-phase survival-related protein, and b1374, a putative transposon resolvase. In the compendium, *ynaE* was highly expressed when Lon protease or YoeB toxin was genetically up-regulated, and when either norfloxacin antibiotic or mussel defensin protein was present. Based on our analysis, YnaE may control a small, specialized stress response network in E. coli.

Overall, we were able to detect a significant (one-tailed *p* value < 0.05) binding motif for 28 out of the 61 transcription factors (Table S3). Of these regulators, 13 had a known motif in PRODORIC (http://prodoric.tu-bs.de/). We compared the predicted motifs for the 13 regulators to all known E. coli motifs. For seven of the 13 MEME-predicted motifs (54%), we identified the known motif in the top ten best matches (Table S3). Some correctly reconstructed regulators made a relatively poor match to their correct motif. In the majority of these six cases, including Lrp (rank = 15) (Figure S3B), this was due to the presence of combinatorial or conditional regulation. For Lrp, two motifs in the database (± leucine) were incorrectly collapsed into one by motif analysis. In other cases, a gene was regulated by multiple transcription factors, and the MEME motif analysis picks up the stronger motif in the sequence, which may not be the motif of the transcription factor we are looking for. This ambiguity in cases of combinatorial regulation is a limitation of motif analysis, because it only looks for statistical enrichment of regions of sequence and does not consider the actual binding properties of the transcription factor.

#### In vivo confirmation of new regulatory interactions.

We performed ChIP with quantitative PCR (ChIP-qPCR) to obtain physical confirmation for many of the regulatory interactions inferred by CLR and to verify that performance estimates based on the known subset of interactions in RegulonDB extrapolate beyond this subset. In particular, we studied three transcription factors (Lrp, PdhR, and FecI) with substantial connectivity in the network mapped by CLR. For each transcription factor, 26–35 operons with at least one inferred interaction were tested by ChIP-qPCR for a total of 93 tested operons (244 genes). We tested 24 known regulatory interactions as a positive control to verify that known interactions were detectable by ChIP for a total of 268 tested interactions.

Network inference results are typically verified for a few hand-picked samples that are studied in detail. Thus, the biological intuition of the experimenter may play a role in the success of a hand-picked verification. RegulonDB provides a way to overcome this problem in E. coli. Because we have no control over the interactions present in RegulonDB, our algorithm performance estimates are unbiased by our selective validation. Likewise, when performing ChIP validation experiments, we wanted to be as unbiased as possible. Although choosing the three transcription factors is a form of hand-picking, the targets tested for those transcription factors were selected in a systematic way, by choosing approximately 30 of the highest-scoring targets of each of the three transcription factors. Choosing this many targets results in many interactions with confidence levels below 20% in our ChIP-qPCR experiments, allowing us to verify that our confidence estimates are reliable across the entire range of precision.

**Figure 5:**
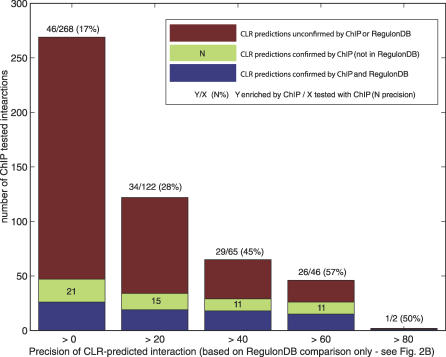
Experimental Validation of Inferred Regulatory Interactions Global precision scores determined with RegulonDB for a set of 268 regulatory interactions were in good correspondence with the local precision scores determined via RegulonDB plus ChIP for three transcription factors. The blue bar indicates inferred interactions that are true positives based on RegulonDB and ChIP. The green bar shows the number of inferred interactions not in RegulonDB that were positive for ChIP, representing 21 new experimentally verified regulatory interactions. The red bar shows inferred interactions that are false positives based on RegulonDB and ChIP.

Figure 5 shows the global, RegulonDB-based precision scores assigned to the ChIP-tested interactions versus the transcription factor–specific precision estimated with RegulonDB plus ChIP, including many interactions for genes not present in RegulonDB. The global precision estimate based on RegulonDB corresponds very well to the local precision estimate based on RegulonDB plus ChIP. Only the two extreme cases of greater than 80% precision and greater than 0% precision deviate from the expected values. The greater than 0% precision interactions yield an inflated ChIP precision, because our choosing of the highest-scoring targets for validation causes this category to be undersampled. Likewise, the greater than 80% precision has only two samples, because interactions of this significance are rare in the dataset, making the ChIP estimate unreliable for this threshold. In total, 21 novel regulatory interactions were confirmed in vivo by this approach, adding to the knowledge of E. coli regulation present in RegulonDB.

#### A combinatorial link between central metabolism and iron transport.

The inferred regulatory network revealed new combinatorial regulation at many promoters. We explored these combinatorial regulation schemes, first across the entire network (Protocol S1) and second by detailed real-time quantitative PCR analysis of the novel PdhR-*fecA* interaction, which is an interaction that links central metabolism to the control of iron import—a link of potential importance in bacterial virulence and stress protection.

The presence of iron is essential for the survival of most organisms, because it plays a critical role in the tricarboxylic acid cycle, in electron transport, in reducing oxygen radicals, in DNA synthesis, and in amino acid synthesis [37]. Iron, however, is scarce in many environments because of the low solubility of its ferric form. Consequently, many organisms have developed elaborate mechanisms for scavenging soluble forms of the element. In E. coli K12, there are six different siderophore receptors, each representing a different chelator that is capable of capturing extracellular iron and converting it to a soluble form that may be transported into the cell [38]. Excess iron can be toxic to cells; iron uptake must therefore be carefully dictated by the need for cellular iron.

**Figure 6:**
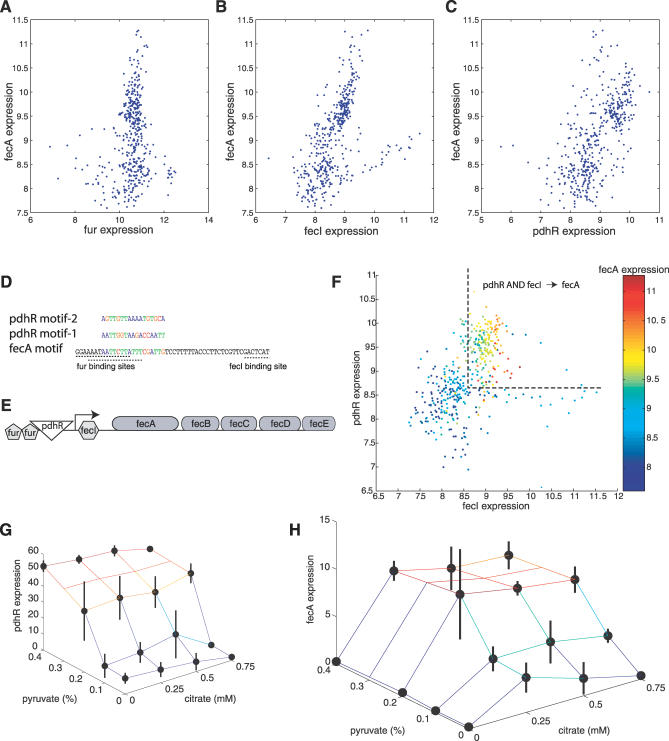
Analysis of the Regulation of the *fecABCDE* Iron Transport Operon All expression data are log_2_ transformed and RNA normalized. (A) Fur shows no correlation to the *fecA* operon, one of its known target operons. (B) FecI shows correlation to its known operon target *fecA* with a bifurcation that suggests combinatorial regulation by another transcription factor. (C) PdhR, a regulator of pyruvate metabolism, is not known to regulate the *fecA* operon. However, their expression values are correlated in the compendium. (D) Alignment of known PdhR binding motifs with *fecA* promoter. The known FecI binding motif is further downstream. (E) A schema of the new proposed regulatory structure of the *fecABCDE* operon. (F) Viewing the expression of *fecA* (the *z*-axis is represented as color changes corresponding to the values on the color bar on the right) as a function of both transcription factors suggests its regulation by FecI and PdhR might be AND-like. (G) *pdhR* expression is highly dependent on the concentration of pyruvate in the media. Expression values exhibit high uncertainty at the threshold pyruvate concentration of 0.2% (represented by vertical error bars), suggesting a bifurcation of cells into high and low expression states. (H) *fecA* expression was measured at 16 concentrations of two chemicals, citrate and pyruvate, known to alter the expression of *fecI* and *pdhR*, respectively. The results further support the hypothesis that *fecA* expression is controlled with AND-like behavior by FecI and PdhR. *fecA* expression exhibits high uncertainty at 0.25 mM citrate and 0.2% pyruvate. As with *pdhR* expression in (G), this high uncertainty may reflect the probabilistic nature of induction near the switching threshold.

*fecABCDE* is an operon that encodes a ferric citrate transporter and plays a central role in the import of cellular iron. Existing literature described only two regulators of *fecABCDE*—FecI and Fur. The Fur regulation is not apparent in the compendium (Figure 6A), while the FecI regulation is clear (Figure 6B). However, the bifurcation of the plot suggests a more complex combinatorial regulation for *fecABCDE*. The CLR algorithm identified PdhR, a pyruvate-sensing repressor and necessary component of the energy transduction cascade, as a possible additional regulator of the *fecA* operon (Figure 6C). We also identified a potential PdhR binding motif in the promoter region of the operon (Figure 6D and 6E). Moreover, in undefined, rich media (Luria-Bertani [LB] with 0.2% glucose), our ChIP results showed a significant enrichment for PdhR-*fecA* binding when judged by a *t*-test (*p* value = 0.004) and a modest enrichment using a nonparametric rank-sum test (*p* value = 0.1).

Inspection of the compendium data suggested that FecI and PdhR might regulate the *fecA* operon using AND-like logic, where both proteins must be activated for expression of the *fecA* operon (Figure 6F). Because PdhR is a repressor that is derepressed upon binding with pyruvate, the gate is NOT (bound PdhR) AND (bound FecI) at the promoter level; self-feedback at the *pdhR* promoter makes the gate appear as *(pdhR)* AND *(fecI)* at the level of mRNA (Figure 6F). To test this hypothesis, we used real-time quantitative PCR to measure the expression level of *fecA* over 16 combinations of pyruvate (to derepress PdhR protein and induce *pdhR* transcription; Figure 6G) and citrate (to activate FecI and induce *fecI*). The *fecA* operon reached its highest levels of induction only when citrate and pyruvate were both present in high concentrations, supporting the hypothesis that full activation of *fecA* is only possible in the presence of derepressed PdhR and activated FecI (Figure 6H).

Such an explicit regulatory link between central metabolism and iron transport has not, to our knowledge, been previously identified in microbes. This link is perhaps not surprising, given that iron is a critical component of several proteins involved in both the tricarboxylic acid cycle (aconitase and succinate dehydrogenase) and electron transport (cytochromes and ferredoxin); the magnitude of carbon/electron flux through the citric acid cycle and electron transport chain thus plays a major role in determining the cellular need for iron. It is possible that an increase in intracellular pyruvate, which is the inducer for PdhR, may signal the need for increased flow through respiratory pathways. This novel role for pyruvate is plausible given that pyruvate serves as a common catabolite for a diverse collection of carbon sources and stands just one enzymatic step away from entering the tricarboxylic acid cycle itself.

### A Platform for Mapping Transcriptional Regulation

This work rigorously assesses the genome-scale performance of multiple network inference algorithms using an experimental compendium of 445 expression profiles and a “gold standard” of known regulatory interactions. Using the top-performing CLR algorithm, we predicted 1,079 regulatory interactions in E. coli with 60% precision or higher (and 426 interactions at 80% precision or higher). ChIP experiments confirmed 21 CLR-inferred regulatory interactions, and real-time PCR analysis combined with the inferred regulatory map suggested that iron regulation and central metabolism are linked at the level of transcriptional regulation. We also showed that CLR could infer an equally precise network map using as few as 60 expression profiles, and our results help to address persistent questions concerning the optimal design of experiments for network mapping based on machine learning (Protocol S1).

In recent years, ChIP techniques, particularly ChIP-Chip, have offered hope for systematic characterization of transcription factor binding in vivo. ChIP is particularly prone to errors in prokaryotes, necessitating a large number of expensive replicates [39]. Moreover, the results are condition-dependent, i.e., inactive transcription factors may not be identified because they may not bind to DNA. Finding the appropriate conditions for ChIP-Chip can be costly and time-consuming, making the comprehensive mapping of microbial transcriptional networks difficult.

By generating a compendium of microarrays, we show that it is possible to infer a high-precision regulatory map and simultaneously obtain rich data on condition-specific regulation. With this conditional regulatory information, we can also make a more informed decision about when a transcription factor might be active in any follow-up ChIP, mass-spectrometry, or real-time PCR experiments.

We suggest that E. coli, a long-standing model organism for the detailed study of small-scale regulatory circuits, can become a valuable model organism for large-scale regulatory network studies, by virtue of the availability of (1) a large and curated set of experimentally determined regulatory interactions; (2) a tested expression compendium; and (3) a reliable platform for the acquisition of additional expression data.

## Materials and Methods

### Microarray profiling.

To explore pathways of particular importance to antibiotic resistance, we assayed 121 conditions using 266 microarrays, including more than 50 genetic perturbations (overexpression or knockout) during norfloxacin-induced DNA damage response, overexpression of the ccdB toxin, and growth to stationary phase on low and high glucose (Table S4).

### Bacterial strains.

Fifty-three E. coli genes of interest were overexpressed in E. coli strain MG1655 (E. coli Genetic Stock Center, CGSC 6300) using a modified pBAD30 vector, pBADx53 [40]. pBADx53 has a low copy SC101 origin of replication, does not contain *araC,* and yields low and consistent levels of expression, generally increasing gene expression 2- to 10-fold above native expression levels. The 53 genes were PCR amplified from MG1655 genomic DNA. A ribosomal binding site was included at the start of the forward primer. The cloned genes were transformed into strain MG1655. Gene deletions were constructed from E. coli strain MG1655 by replacing the coding sequence from start codon to stop codon [41]. Gene deletion strains and overexpression plasmids were confirmed by DNA sequencing. Note that there is a known deletion around the *fnr* gene in strain CGSC 6300 [42].

### Steady-state experiments.

Gene deletion strains and pBADx53 overexpression strains were grown in 96 square-well plates containing 1.6 ml LB (Miller). LB media for the overexpression strains contained 0.125% arabinose to induce cloned gene expression and appropriate antibiotics to maintain the plasmid. Plates were incubated at 37 °C with shaking at 300 revolutions per minute (rpm). DNA damage responses were induced by growing perturbation strains for 3 h in norfloxacin (25–100 ng/ml). Cells were harvested when the optical density (OD600) for the cultures was between 0.25 and 0.40.

### Time-course experiments.

For an antibiotic time-course experiment, cultures were grown in 250-ml flasks at 37 °C with shaking at 250 rpm. Each culture was grown in 75 ml of LB to 0.4 OD600. DNA damage was induced with 10 μg/ml of norfloxacin. Samples were taken before and 12, 24, 36, and 60 min after the addition of norfloxacin. For the glucose time series, E. coli EMG2 were diluted 1:1,000 into 150 ml LB (Miller) in 1-l baffled flasks supplemented with 0.2% or 0.4% glucose. Samples were taken 2.5, 3, 3.5, 4, 4.5, 5, 5.5, 6, and 8 h post-incubation. To examine the effect of overexpression of the F-plasmid encoded toxin CcdB, we used a plasmid-borne riboregulation system that enables precise control of gene expression through highly specific RNA-RNA interactions [43]. A riboregulation system overexpressing LacZ was included as a control. Cells were diluted 1:1,000 in 50 ml LB (Miller) with appropriate antibiotics to maintain the plasmid. Samples were taken immediately before induction and then 30, 60, and 90 min after induction of CcdB or LacZ expression.

### Preparation of RNA and hybridization.

RNA was prepared using Qiagen RNeasy kits (Valencia, California, United States). For time-course experiments, cultures were immediately added to 2 volumes of Qiagen RNAprotect reagent. For steady-state experiments, 1.5-ml cultures in multiwell plates were centrifuged at 3,000 g for 5 min at 4 °C. Media was poured off, and 500 μl of RNA protect was then immediately added to each cell pellet. cDNA was prepared and hybridized to the Affymetrix Antisense2 microarrays according to the standard Affymetrix prokaryotic sample and array processing protocol.

### External data.

A literature search was performed to locate microarray datasets to expand the phenotypic diversity of the compendium. Preference was given to larger datasets (>10 chips). In total, raw Affymetrix CEL files for 179 microarrays were compiled from nine different publications (Figure 1 and Table 1). These microarrays assayed 68 conditions including pH changes, growth phases, antibiotics, heat shock, different media, varying oxygen concentrations, numerous genetic perturbations, several carbon sources, and nitrate.

### Microarray normalization.

Raw probe intensities were normalized to gene expression levels using MAS5 (Affymetrix), RMA [44], GCRMA [45], and Dchip PM [46]. All methods were run using the default parameters. For GCRMA, the ad hoc algorithm was used instead of the full empirical Bayes method due to memory constraints arising from the size of the dataset. In our experience, RMA was the single best normalization method of the four that we tried, and the results presented for all algorithms use this normalization unless otherwise indicated.

### Data availability.

The 212 Affymetrix CEL files generated from our own experiments have been submitted to Gene Expression Ominbus (GEO), the NCBI microarray database (http://ncbi.nlm.nih.gov/geo/). Raw and normalized data for all 445 microarrays are available at M^3D^ (http://m3d.bu.edu). M^3D^ provides a web interface for visualizing heat plots, histograms, and scatterplots for any subset of the genes and experiments in the compendium using any of the four normalization methods mentioned above. It also allows the download of the CLR-inferred network in several different formats at both 60% and 80% precision.

### Network inference algorithms.

We adapted several existing methods suitable for whole-genome network mapping from expression data. These methods were relevance networks [29], ARACNe [2], and Bayesian networks [7]. In addition, we developed a novel method, CLR, which constitutes a background-corrected approach to relevance networks. All tested algorithms are available at the M^3D^ Web site.

Relevance networks, ARACNe, and CLR use mutual information as a metric of similarity between the expression profiles of two genes. The mutual information for two discrete random variables *X* and *Y* is defined as:

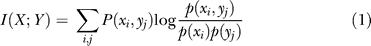
where *P(x_i_)* is the probability that *X = x_i_*. For genes, *X* and *Y* represent a transcription factor and its potential target gene, and *x_i_* and *y_i_* represent particular expression levels. In the case of continuous random variables, the summations over *X* and *Y* are replaced by integrals. To compute mutual information, we used a B-spline smoothing and discretization method, except for comparison testing with the original algorithms, as noted below [47]. We provide a MatLab (http://www.mathworks.com/) interface to the Daub et al. [47] B-spline mutual information estimation code library at the M^3D^ Web site. All mutual information values were computed using 10 bins and third order B-splines.

The performance of the ARACNe, Bayesian, and linear regression network algorithms improved if we inferred four networks, each network calculated from the compendium data that was normalized with a different one of the four normalization methods. For each edge, we then computed the mean of scores resulting from the four networks (Protocol S1). This averaging approach did not improve the results for relevance networks or CLR.

### Relevance networks.

The relevance networks algorithm identifies a potential biological association as any regulator-target gene pair with a mutual information score between their expression profiles that is above a set threshold. Although originally intended as a form of clustering, we applied the algorithm to network inference by only keeping associations between transcription factors and genes. The original relevance networks algorithm generated one network at one threshold; for the algorithm comparison, we applied a range of thresholds to generate a precision versus recall curve.

### ARACNe.

We obtained the authors' original implementation of the ARACNe algorithm [30] for Linux (http://amdec-bioinfo.cu-genome.org/html/aracneregistration.html). As recommended in the algorithm documentation, we restricted the tolerance threshold, τ, to between 0 and 0.15 and sampled this parameter evenly. As used by the ARACNe authors [2] and recommended in the distribution, we built mutual information tables using the “fast” method (sliding window/“naïve” estimator).

In the original ARACNe algorithm, an edge is pruned when it falls outside of the tolerance threshold of every interaction triangle formed by applying the data processing inequality. We created a modified implementation of the ARACNe algorithm that uses the B-spline mutual information estimate and a probabilistic threshold to improve performance (Protocol S1). For the probabilistic threshold, we computed the frequencies of keeping each edge, on the basis of all of the data processing inequality comparisons in which it participated, and we pruned the network using these frequencies. We computed the mutual information matrix using every probe set (as we also did for CLR), including the intergenic regions, to make probabilistic scores using the largest possible distribution.

### CLR.

Bias from uneven condition sampling, upstream regulation, and inter-laboratory variations in microarrays complicate network inference, because indirect regulatory influences and direct (physical) regulatory interactions may not be easily distinguishable from their expression profiles. Our new algorithm, CLR, increases the contrast between the physical interactions and the indirect relationships by taking the network context of each relationship into account. CLR uses the local network context to compute a significance estimate for any statistical metric of similarity between gene expression profiles. We have shown that CLR performs best with mutual information but also performs well with Pearson correlation (Protocol S1).

Like relevance networks and ARACNe, CLR uses the matrix of mutual information values between all probe sets on the Affymetrix array. The CLR algorithm estimates a likelihood of the mutual information (MI) score for a particular pair of genes, *i* and *j*, by comparing the MI value for that pair of genes to a background distribution of MI values (the null model). The background distribution is constructed from two sets of MI values: {MI*_i_*}, the set of all the mutual information values for gene *i* (in row or column *i*), and {MI*_j_*}, the set of all the mutual information values for gene *j* (in row or column *j*) (Figure 2A). Because of the sparsity of biological regulatory networks, most MI scores in each row of the mutual matrix represent random background MI (e.g., due to indirect network relationships). We approximate this background MI as a joint normal distribution with MI*_i_* and MI*_j_* as independent variables, which provides a reasonable approximation to the empirical distribution of mutual information (Figure S4). Thus, the final form of our likelihood estimate becomes 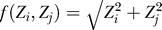 , where *Z_i_* and *Z_j_* are the *z*-scores of MI*_ij_* from the marginal distributions, and f(*Z_i_*,*Z_i_*) is the joint likelihood measure. We experimented with other approximations of the background MI distribution, including the generalized extreme-value distribution, the Rayleigh distribution, and the Gaussian kernel density estimator (empirical), always achieving similar results and sacrificing speed of execution for the more expensive distribution fits.

Our method of estimating the likelihood of mutual information differs from the conventional ways of estimating significance. For example, both the analytical Roulston metric of significance [33] and shuffling [47] calculate the statistical significance given a random model of the interaction in question (Protocol S1). In contrast, CLR calculates the likelihood of mutual information given the observed network context, which consists of the background distribution formed by the mutual information for all possible incoming and outgoing edges for one gene in the network.

### Bayesian networks and linear regression networks.

Unlike the pairwise algorithms described above, Bayesian networks exhaustively or heuristically search through the multivariate space of possible graphs (i.e., regulatory networks), scoring each, and keeping either the best-scoring network or a network constructed by averaging over all the searched graphs and weighting them by their score. This multivariate approach comes at a computational cost. None of the publicly available Bayesian network learning software that we tried were designed to infer a network of the size we attempted with this study, nor would they run to completion. Those that did not crash due to memory problems were unable to generate a network after several weeks of computational time.

For computational tractability, we wrote a Bayesian network algorithm that implemented a series of constraints. Every gene was restricted to having at most two regulators, and interactions were only allowed between transcription factors and genes. We tested several scoring functions for the algorithm: discrete (two-state, genes are OFF or ON), linear, logistic, polynomial approximation, and hill function. Scores for the linear function were estimated with linear least-squares fitting. Scores for nonlinear functions were estimated with nonlinear least-squares fitting using the Levenberg-Marquardt algorithm. All scores were adjusted for the number of parameters using Bayesian information criterion. Of the tested scoring methods, the linear function offered the best balance between speed and quality of reconstruction.

We used a model averaging procedure to score the likelihood of each edge in the regulatory network. Transcription factor/gene interactions were exhaustively scored as follows. For a particular gene *A*_i_ in a regulatory network allowing only one regulator per gene, the likelihood of being regulated by a given transcription factor *B*_j_ was calculated as 
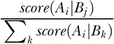
 where *k* is indexed over all identified transcription factors. This function was generalized to the case of two transcription factors per gene to account for the scoring of the same transcription factor/gene interactions in multiple network models.

Transcription factor/transcription factor interactions were initially scored using a different approach. We sampled directed acyclic graphs of the transcription factor only network using Metropolis Coupled Markov Chain Monte Carlo. We then applied the averaging approach above for the sampled networks. However, we found that in practice, both the speed and precision of the algorithm improved if the transcription factors were scored in the same way as for transcription factor/gene interactions (Protocol S1). This resulted in networks that were no longer directed acyclic graphs, and thus the algorithm was no longer a true Bayesian network and is referred to as a linear regression network.

### Construction of the reference set of interactions.

We obtained all known regulatory interactions catalogued in RegulonDB version 4 (http://regulondb.ccg.unam.mx/html/Data_Sets.jsp) [27]. Of the interactions, 2% could not be matched to probe sets on the expression array or had only self-regulation. We removed these interactions from the reference set. In addition, several genes thought to be E. coli regulators were present in our reference set but were not known to regulate anything within it. Therefore, we also removed these genes from all inferred networks when estimating the algorithm's performance, leaving 3,216 non-self interactions among 1,211 genes as the reference network. We also obtained a list of 328 putative and known transcription factors from RegulonDB.

### Measurement of algorithm performance.

To evaluate the performance of all algorithms, we constrained the resulting network maps to include only the genes available in our RegulonDB control set. We computed the precision and recall of the inferred networks by comparing the inferred network to the reference network. Precision is the fraction of predicted interactions that are correct [*TP*/(*TP* + *FP*)], and recall is the fraction of all known interactions that are discovered by the algorithm [*TP*/(*TP* + *FN*)], where *TP* is the number of true positives, *FP* is the number of false positives, and *FN* is the number of false negatives. Precision and recall were computed over a range of pruning thresholds; interactions with scores below the pruning threshold were removed from the inferred network. Both precision and recall are reported as percentages.

## Supporting Information

Figure S1 The Transcriptional Regulatory Map Inferred by CLR with an Estimated 80% Precision The precision of the network is obtained by measuring the percentage of correctly inferred edges (blue lines) out of all the predicted edges for genes with known connectivity (blue lines and green lines). The green edges represent a mixture of false and novel predictions, making 80% an underestimate. The red edges are to genes without a previously identified regulator or from regulators without a previously known target. Transcription factor nodes are colored light gray.
(162 KB PDF)

Figure S2 Transcription Factor Recall Transcription factors, with at least two inferred interactions (blue bar), have high recall (47% on average) of the their known targets (green bar versus red bar); this suggests that when the transcription factors in the compendium are perturbed by the appropriate condition, much of that transcription factor's regulon is correctly identified.
(88 KB PDF)

Figure S3 Motifs Detected for Four of the Transcription Factors with Five or More Target Operons (A) The canonical LexA regulatory motif was detected in the promoters of eight out of the 13 genes inferred to be LexA targets.
(B) The canonical Lrp regulatory motif was also detected with high significance.
(C) A novel motif was found for YnaE, a transcription factor that may play a role in the regulation of a prophage or DNA repair.
(D) YmfN, another prophage-related transcription factor with no known regulatory targets, had a strong motif conserved in all of its predicted targets.
(153 KB PDF)

Figure S4 Estimating the Distribution of Mutual Information The distribution of mutual information for both genes of a potential regulatory interaction is used to estimate the significance of mutual information. The distribution of mutual information for one gene, *lexA,* illustrates different types of fit. Normal fit, while not the best approximation to the empirical distribution, penalizes the distal network neighborhood.
(661 KB PDF)

Table S1 Functional Enrichment of YmfN Targets (34 KB DOC)

Table S2 Functional Enrichment of YnaE Targets (25 KB DOC)

Table S3 *z*-Scores of Motifs for Transcription Factors in the 60% Precision Network with ≥5 Predicted Operon Targets (194 KB DOC)

Table S4 The Clustered Microarrays of the E. coli Compendium (1.2 MB DOC)

Protocol S1 Click here for additional data file. Additional Supporting Methods, Results, Figures, and Tables
(809 KB DOC)

## Abbreviations

ChIP: chromatin immunoprecipitation
CLR: context likelihood of relatedness
LB: Luria-Bertani
M^3D^: Many Microbe Microarrays database
MI: mutual information
