# Supplementary material for: Large-Scale Mapping and Validation of Escherichia coli Transcriptional Regulation from a Compendium of Expression Profiles
Source: PLoS Biol. 2007 Jan 9;5(1):e8. doi: 10.1371/journal.pbio.0050008 (PMC1764438; doi:10.1371/journal.pbio.0050008)
Supplement: Supplementary file 1 [file pbio.0050008.sg001.pdf]

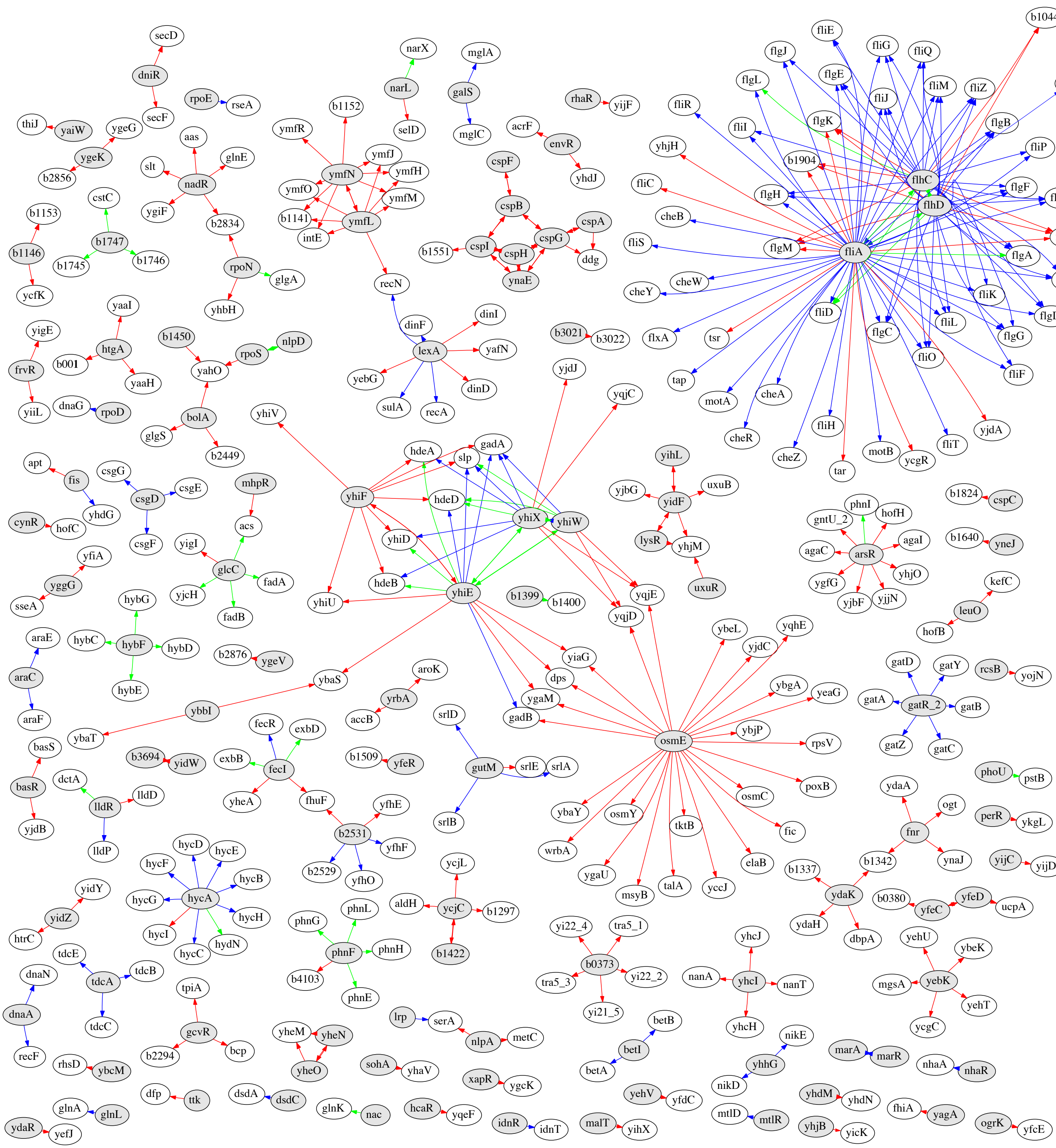

Figure S1. The transcriptional regulatory map inferred by CLR with an estimated 80% precision.

The precision of the network is obtained by measuring the percentage of correctly inferred edges (blue lines) out of all the predicted edges for genes with known connectivity (blue lines and green lines). The green edges represent a mixture of false and novel predictions, making 80% an underestimate. The red edges are to genes without a previously identified regulator or from regulators without a previously known target. Transcription factor nodes are colored light gray.
