# Supplementary material for: Large-Scale Mapping and Validation of Escherichia coli Transcriptional Regulation from a Compendium of Expression Profiles
Source: PLoS Biol. 2007 Jan 9;5(1):e8. doi: 10.1371/journal.pbio.0050008 (PMC1764438; doi:10.1371/journal.pbio.0050008)
Supplement: Supplementary file 2 [file pbio.0050008.sg002.pdf]

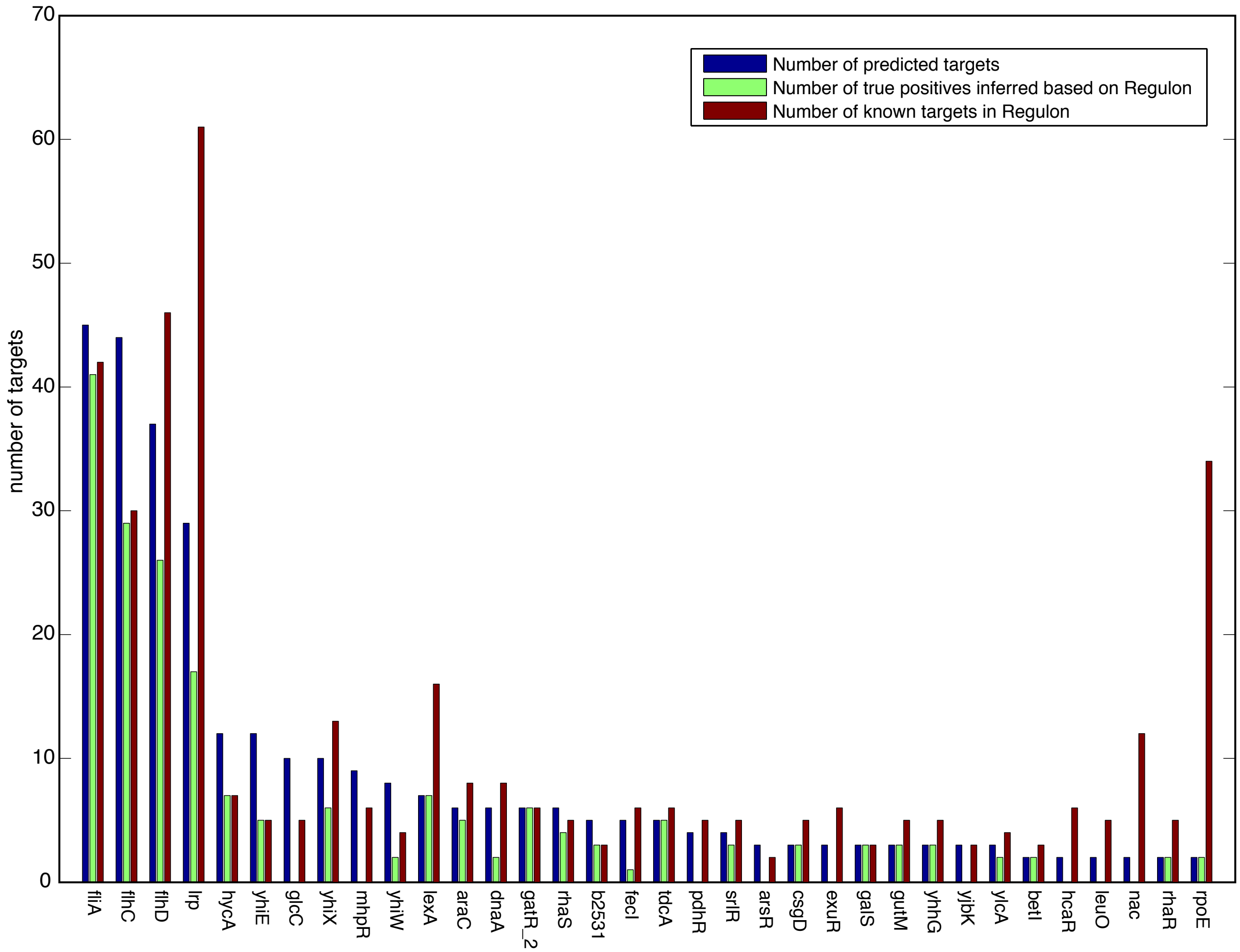

Figure S2. Transcription factor recall

Transcription factors, with at least two inferred interactions (blue bar), have high recall (47% on average) of the their known targets (green bar –vs- red bar); this suggests that when the transcription factors in the compendium are perturbed by the appropriate condition, then much of that transcription factor's regulon is correctly identified.
