# Supplementary material for: Large-Scale Mapping and Validation of Escherichia coli Transcriptional Regulation from a Compendium of Expression Profiles
Source: PLoS Biol. 2007 Jan 9;5(1):e8. doi: 10.1371/journal.pbio.0050008 (PMC1764438; doi:10.1371/journal.pbio.0050008)
Supplement: Supplementary file 3 [file pbio.0050008.sg003.pdf]

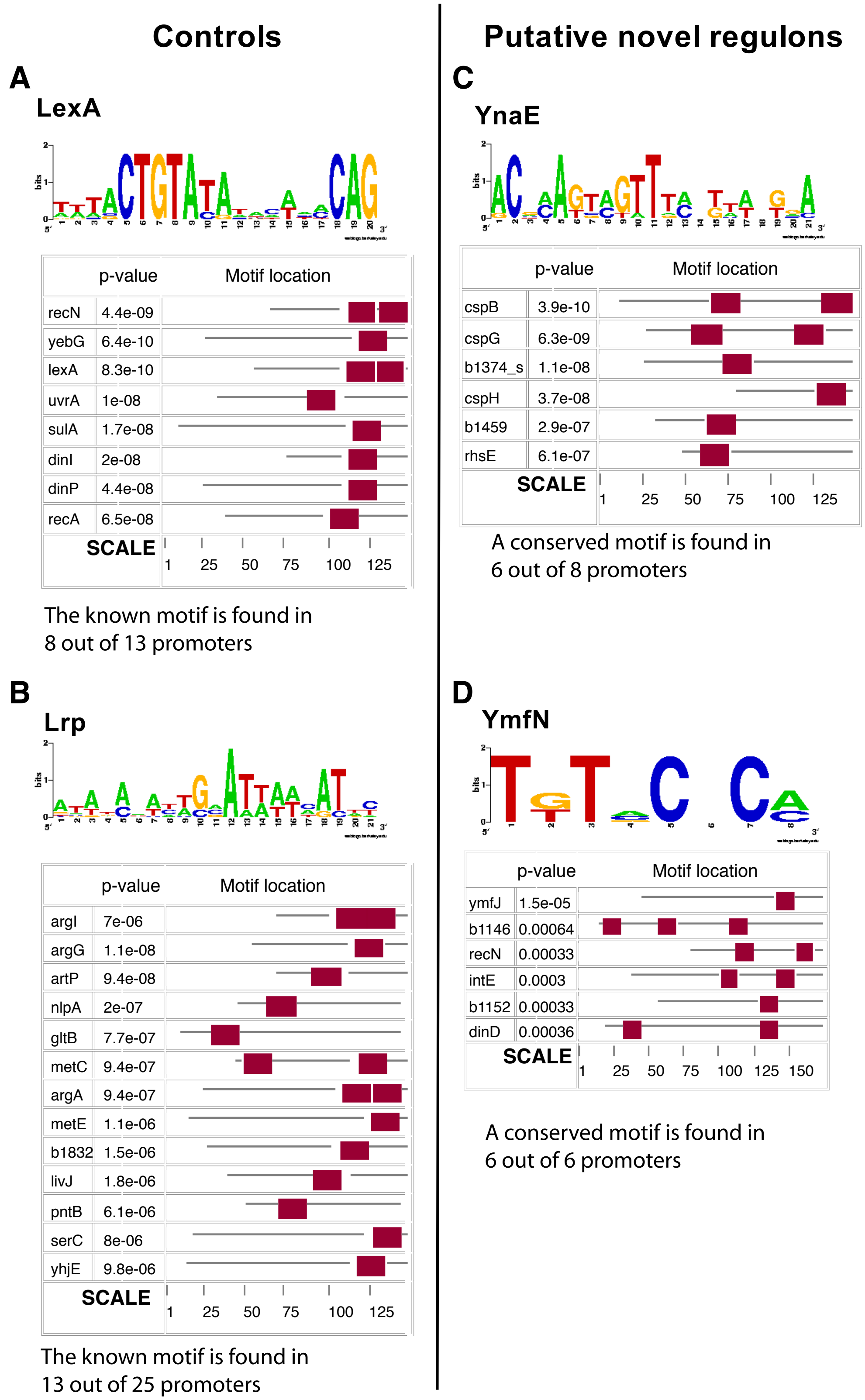

Figure S3. Motifs detected for four of the transcription factors with five or more target operons.

(A) The canonical LexA regulatory motif was detected in the promoters of 8 out of the 13 genes inferred to be LexA targets.
(B) The canonical Lrp regulatory motif was also detected with high significance.
(C) A novel motif was found for YnaE, a transcription factor that may play a role in the regulation of a prophage or DNA repair.
(D) YmfN, another prophage-related transcription factor with no known regulatory targets, had a strong motif conserved in all of its predicted targets.
