# Supplementary material for: Large-Scale Mapping and Validation of Escherichia coli Transcriptional Regulation from a Compendium of Expression Profiles
Source: PLoS Biol. 2007 Jan 9;5(1):e8. doi: 10.1371/journal.pbio.0050008 (PMC1764438; doi:10.1371/journal.pbio.0050008)
Supplement: Supplementary file 4 [file pbio.0050008.sg004.pdf]

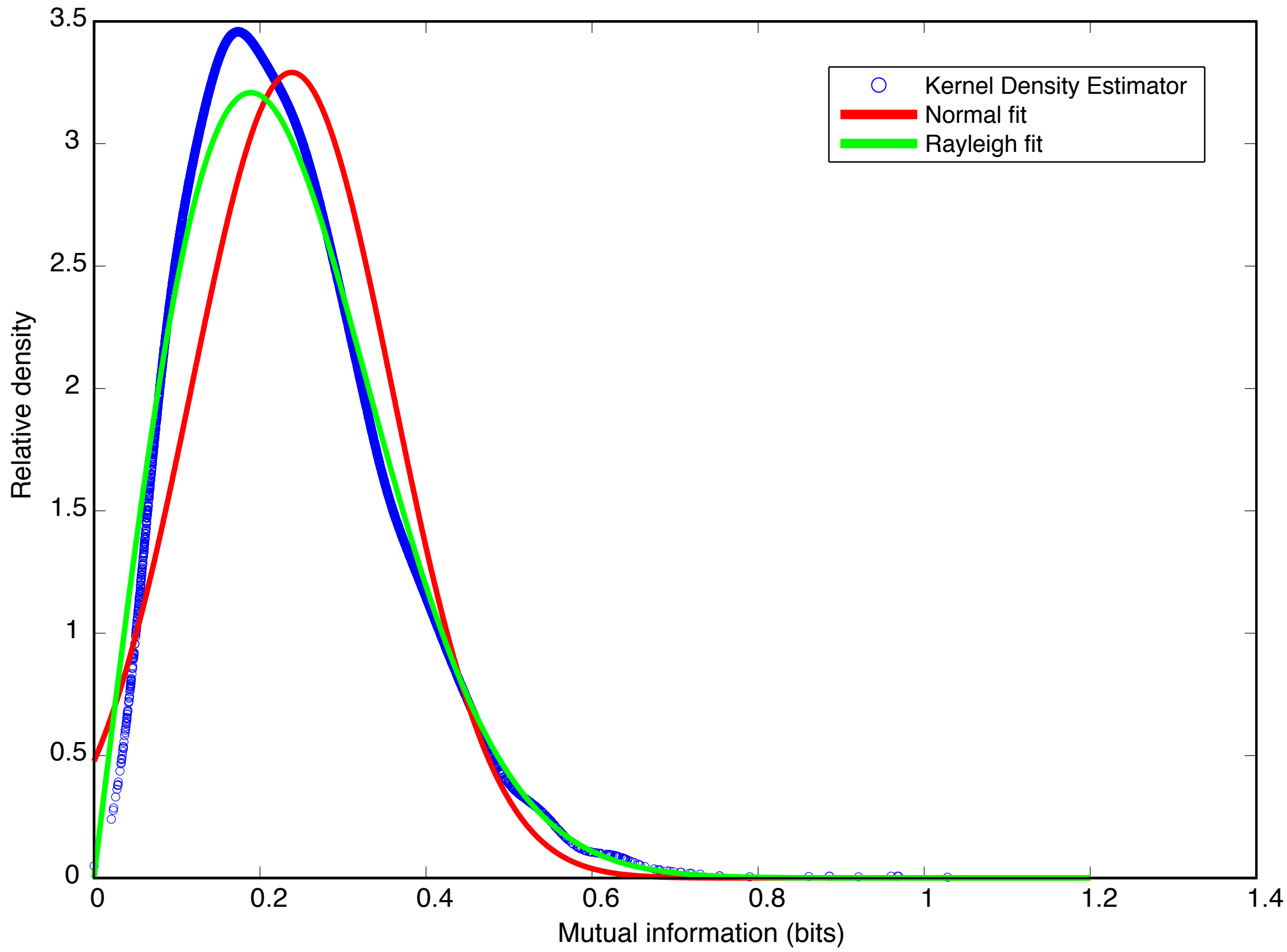

Figure S4. Estimating the distribution of mutual information.

The distribution of mutual information for both genes of a potential regulatory interaction is used to estimate the significance of mutual information. The distribution of mutual information for one gene lexA illustrates different types of fit. Normal fit, while not the best approximation to the empirical distribution, penalizes the distal network neighborhood.
