# Supplementary material for: Large-Scale Mapping and Validation of Escherichia coli Transcriptional Regulation from a Compendium of Expression Profiles
Source: PLoS Biol. 2007 Jan 9;5(1):e8. doi: 10.1371/journal.pbio.0050008 (PMC1764438; doi:10.1371/journal.pbio.0050008)
Supplement: Supplementary file 5 [file pbio.0050008.sd001.doc]

**Protocol S1:**

**Large-scale computational mapping and experimental validation of *Escherichia coli* transcriptional regulatory interactions from a compendium of expression profiles**

Jeremiah J. Faith, Boris Hayete, Joshua T. Thaden, Ilaria Mogno, Jamey Wierzbowski, Guillaume Cottarel, Simon Kasif, James J. Collins, and Timothy S. Gardner

**Summary**. This supplement provides additional figures and results on the comparison of network algorithms, additional discussion of our conclusions regarding optimal experimental design for network inference, and additional results on global properties of the inferred networks. We also describe methods not provided in the main text.

**Supplementary Results**

**Verification of array data normalization and consistency**

The algorithms applied in this study require the consolidation of results from disparate sources including different experimenters and different laboratories. To minimize the impact of experimentally induced error, we used only expression data collected on a single platform, the Affymetrix Antisense2 microarray. Nevertheless, we evaluated whether such data could be reliably integrated and still preserve quantitative information on expression levels of transcripts in *E. coli* across many conditions.

After normalization, our analysis shows that coordinated expression responses in the compendium are observed regardless of the laboratory or individual running the experiment, verifying the consistency of the array platform and normalization procedure. Figures S5a, S5b, and S6b show relationships between different transcription factors and their known targets that are characteristic of the data in our *E. coli* microarray compendium. The wide range of expression levels for the genes presented in Figure S5 result from the cell’s response to the different environmental and genetic perturbations assayed in the compendium. In each plot, the expression level of the regulated gene increases with the expression level of its regulator. For example, Figure S5a shows the expression levels of *recA*, an important gene involved in the SOS-response to DNA damage, and of *lexA*, the primary transcription factor of the SOS-response [1]; each gene is highly expressed only when a DNA-damaging agent is present in the growth media. A similar situation occurs for the switch-like arabinose-induced response shown in Figure S5b. Although some lab-to-lab variation is observed in the expression response under similar conditions, we conclude that the dominant changes are due to biological responses not to interlab variations. Thus we can reliably combine the data from disparate sources into a single compendium for quantitative analysis.

**Comparison of network algorithm variants**

In Figure S7, we present a comparison of the original published versions of these algorithms, as well as modified versions of the algorithms. We developed the modified versions of the algorithms in order to improve their performance on the *E. coli* data.

***Summary of tested algorithms***

We study two major groups of network inference algorithms. In the first group, which is represented by multivariate approaches including Bayesian networks and regression-based methods, the algorithm considers one or more potential regulators for each gene and the functional form of that regulation. Although this class of algorithms directly addresses the problem of determining logic gates and combinatorial regulation, it creates a combinatorial optimization problem that quickly becomes infeasible to compute for large networks. To make the problem computationally tractable, we allowed at most two regulators per gene when applying Bayesian or regression network algorithms (Figure 2b and Figure S7a). The linear regression network approach slightly outperforms the Bayesian network approach because the regression approach allows cycles, of which there are several in RegulonDB. Both methods perform significantly better than random.

The second group of algorithms we tested takes a pairwise approach where each potential interaction between a regulator and a target is considered independently. These methods do not explicitly model the functional form of a combinatorial interaction, but they are more computationally tractable and have no problems scaling to genome-sized networks. In this group we tested relevance networks, ARACNe, and CLR (discussed in main text).

By choosing a high mutual information threshold, relevance networks algorithm achieves precision levels near 90% and obtains reasonable sensitivity (Figure 2b). We also found that Relevance networks constructed using correlation (specifically correlation-squared) as a metric of dependency between two variables outperform networks constructed using mutual information (Figure S7b). This occurs, in part, because mutual information depends on the entropy of the gene pair. Normalization of the mutual information to the minimum entropy of the pair improves the performance (Figure S7b).

One difficulty for relevance networks, and inference algorithms in general, is distinguishing causality from correlation. The ARACNe algorithm attempts to address this problem by applying the Data Processing Inequality to the relevance network to prune away indirect regulatory influences [2,3]. This method was successfully applied to microarray data from human B-cell populations to infer known and novel regulatory interactions, particularly those related to the proto-oncogene *MYC* [4]. We developed a probabilistic ARACNe that outperforms the original and several other variants of ARACNe (Figure S7c) on the *E. coli* compendium, but no version of ARACNe performed as well as the simpler relevance networks algorithm (Figure 2b). The DPI pruning method that underlies ARACNe and probabilistic ARACNe preferentially eliminates direct feedback and feedforward loops. However, such loops are common in microbial regulatory networks [5]. Thus the DPI pruning approach places unrealistic constraints on the resulting regulatory network, which likely accounts for its diminished performance.

Below we provide further rationale for development and implementation of algorithm variants tested in this study.

***Bayesian networks and linear regression networks***

We applied several published Bayesian network algorithms [6-8] to the full *E. coli* compendium. However, none of the algorithms we tested were able to complete their computations due to memory limitations, or run-time errors resulting from the large scale of the data set. These problems are likely due the combinatorial complexity inherent in Bayesian network analysis approaches. To address this we implemented our own Bayesian network algorithm, using a multivariate linear model of combinatorial regulatory interactions, and limited the in-degree of the inferred network to two in order to maintain computational tractability. We also implemented a linear regression network that determines the regulators of each gene independently, and did not assemble the resulting network into a globally optimally network. Therefore the linear regression network, unlike the Bayesian network, permitted the inference of cycles. Like the Bayesian network algorithm, the linear regression network used a multivariate linear model of combinatorial interactions, and also limited the in-degree of the inferred network to two.

The linear regression network algorithm obtained higher performance than the Bayesian network algorithm, presumably because it permitted cycles (Figure S7a), although the resulting network was no longer a rigorous a probabilistic description of the network. Performance improved for the linear regression network and the Bayesian network when we applied each algorithm four times, each time using data normalized with a different one of the four methods, and then averaged the resulting networks (Figure S7a).

***Relevance networks***

For relevance networks, we found that using correlation [9] improved the performance over the original method, which used mutual information (Figure S7b) [10]. This occurs, in part, because mutual information depends on the entropy of the gene pair. Normalization of the mutual information to the minimum entropy of the pair improves the performance (Figure S7b). Knowledge of operons provided slight boosts in recall for certain precision ranges for relevance networks (Figure S7b blue curve; see the CLR section below for the method for using knowledge of operons).

***ARACNe***

With ARACNe, we considered whether it was more effective to apply the DPI pruning after applying the constraint allowing only transcription factor/gene interactions. We found that application of DPI pruning after the constraint lowered performance of the algorithm (data not shown). Thus, for all results presented here, we applied the DPI pruning *before* applying the transcription factor constraint.

Using a spline-based estimation of mutual information improved the performance relative to the original ARACNe (Figure S7c magenta) but did not allow higher recall. Performance improved further by incorporating a probabilistic data processing inequality (DPI). And like the Bayesian network algorithm, ARANCe performance improves when it is run separately on data normalized by four different microarray normalization algorithms and the resulting four networks are averaged.

***CLR***

CLR’s background correction procedure allows determination of a gene-specific significance of mutual information. Other gene-specific background correction approaches have been proposed previously, including the analytical Roulston method [11] and the empirical shuffling approach, which builds the background distribution for a given mutual information score by randomly permuting the two genes’ expression profiles many times. Though gene-specific, the Roulston and shuffling methods are not context-dependent; they compute the significance of mutual information using only the data for a particular pair of genes. In contrast, CLR computes the significance of mutual information by assembling a background distribution from the mutual information scores of all other microarray probe sets in the compendium.

CLR outperforms the Roulston approach (Figure S7d). We do not show results based on the shuffling method, as it was too computationally demanding to achieve the necessary number of permutations for a large network. Nevertheless, we confirmed that the Roulston metric and the shuffling approach converge to similar p-values and overall performance (by running 30 shuffles on a test network of 1211 genes).

The CLR algorithm was applied using both un-normalized and minimum-entropy-normalized mutual information (see the Relevance networks section above). Unlike relevance networks, CLR showed only modest improvement when mutual information was normalized (Figure S7d red), suggesting that the CLR procedure itself normalizes mutual information.

In relevance networks, correlation performed better than mutual information at high sensitivities. Thus, we also applied the CLR background correction method to correlation-based relevance networks. However, mutual information-based CLR outperformed correlation-base CLR in all cases.

Two procedures were tested to determine if prior knowledge of operon structure would improve performance. In the first method, we grouped genes into operons and gave each gene in the operon the median CLR score of the genes in the operon. In the second method, the genes grouped into operons were assigned the maximum CLR score of the genes in the operon. The median CLR score operon method produced negligible improvement (Figure S7d green). The maximum CLR score operon method slightly boosts recall across almost the entire range of precision values (Figure S7d blue). This operon-based performance boost is only possible if operon structure is known *a prior*. For most genes and most novel organisms, operon structure is not known and may be condition-dependent. Thus, we used the CLR without operon data for all further analysis in this study.

**Experimental Design for Network Inference**

Our compendium was not purpose-built for large-scale network inference. It contains conditions reflective of the interests of the contributing laboratories. However, by determining the conditions and factors in these data that are most informative to network inference, we may gain insight into the design of future microarray compendiums constructed with the intent of inferring the largest, most precise regulatory network with the fewest microarrays.

***Diversity of the compendium influences network inference recall***

We expected the number and phenotypic diversity of expression profiles in the compendium to significantly impact the recall of the CLR algorithm. To test the influence of these factors on the inferred network, we identified the smallest set of microarrays sufficient to reconstruct a network equal in recall and precision to a network constructed with all 445 arrays. We used clustering to select the most dissimilar subset of expression profiles from the compendium and measured the performance of the CLR algorithm on this subset. We also analyzed subsets of randomly chosen expression profiles and sets of the most similar profiles.

As expected, the most dissimilar set provided the best performance using the fewest profiles. Using this set, only 60 profiles were required to infer the network with performance equal to that obtained with the entire 445 profiles in the data set (Figure 2c). Each of the 60 conditions is selected from a different cluster where each cluster represents groups of experiments from related environmental conditions (Table S4). Thus, the 60 most diverse conditions are a sufficient representation of the entire dataset. Replication of samples and in-depth sampling of related conditions do not substantially improve the results, as the remaining arrays provide mostly redundant information that does not dramatically increase or decrease performance

Network inference experimental designs have used environmental perturbations [4,12], genetic perturbations [13-18], or some combination of the two [19,20]. Our results suggest that profiling gene expression in diverse environmental conditions relevant to an organism’s life cycle may be the most efficient strategy to systematically map transcriptional regulatory networks in microbes.  Generally, large environmental perturbations, like media changes and addition of drugs, appear to be more informative to our regulatory pathway inference algorithm than genetic perturbations, such as gene overexpressions and knockouts.  Genetic perturbations can be valuable, but they often produce few changes in expression because the scope of their influence will depend on network topology, redundancies in the network and may be condition dependent. Environmental perturbations, on the other hand, are more likely to produce rich expression responses because they often invoke changes in multiple and overlapping pathways.

Additional support for these conclusions is provided by the examples in Figure S5, which show that regulatory relationships become detectable as soon as samples of the relevant condition are added to the compendium. The figures show that LexA targets are only detectable when SOS response conditions are sampled and AraC targets are detectable only when arabinose-metabolizing conditions are sampled. The Lrp regulon (Figure S6b) provides an additional example. When using only those profiles sampled from cells grown in rich (LB) media, Lrp (a transcription factor that is highly expressed in minimal media) had no inferred targets. Most of the known Lrp targets had a weak, incorrect correlation to LexA (Figure S6a inset), but these correlations were correctly eliminated by CLR. Only when the arrays from minimal media were combined into the same dataset did the CLR algorithm successfully identify the targets of Lrp (Figures S6a, S6b, 3, and S3b). This suggests that the expression diversity is of primary importance of identifying regulatory interactions. It also indicates that a network map can be built incrementally by successively profiling the expression of an organism in un-sampled or undersampled physiological states.

We also examined the impact of using time-series on the algorithm performance. Among the 445 microarrays, there are 36 arrays (including replicates) with time-points 30 minutes apart and an additional 24 microarrays with close time-points (12-24 minutes apart). We note that none of the tested algorithms explicitly accounted for dynamic expression measurements (all assumed steady-state data). Thus, we expected that exclusion of the time-series might positively impact our results. However, we applied the CLR algorithm to the compendium with all time-series data removed and found that the performance of the algorithm decreased. To identify what aspect of the time-series data contributed most to network inference, we analyzed the minimal set of 60 experiments needed to reconstruct the network. These experiments generally contained only the first and last time points in each time series, presumably representing extremes of the response. This result again supports the conclusion that phenotypic diversity is most important for network inference.

Note, it is possible that intervening time-points contain information useful for network inference, but because the algorithms do not account for dynamic changes, they cannot benefit from such information. Approaches such as Dynamic Bayesian Networks (DBNs) [3] or smoothing approaches [21,22], might make better use of time-series information. A publicly available DBN algorithm was applied to the data, but did not successfully execute due to problems with the scale of the dataset. Smoothing approaches were not applied due to the ambiguity in selecting smoothing parameters, and the potential for introducing data artifacts in undersampled temporal data.

***Optimizing experimental design for network inference***

How might appropriate conditions be identified for testing? Analysis of an organism’s natural environment provides a rich source of potential test conditions. For example, the natural environment of *E. coli*, the gut, provides a vast set of potential perturbations in the form of food sources, bile, immune factors, antimicrobial peptides, and secreted factors from other microbes that have only recently begun to be studied in the literature [23-26]. Underrepresented conditions in the compendium may also be identified based on the GO function categories with the greatest number of unperturbed transcription factors (Table S5) or genes (Table S6) in the current compendium.

Such a broad approach to sampling the physiology of a microbe presents an experimental challenge because an unmanageable number of conditions are conceivable. Which ones should be sampled, and in which combinations, to obtain the most informative data set? A practical solution to this problem may be found in the statistical design of experiments pioneered 60 years ago by Ronald Fisher. Fisher’s approach addressed the question of how to obtain reliably the most information with the fewest experiments [27,28]. Studies employing factorial designs, fractional factorial designs, or more recent Bayesian designs, are already commonplace in industrial research and optimization. These or similar methods have had proponents in the field of microarrays [29,30], but have received little attention in larger microarrays studies. These designs should make the generation of a compendium covering the phenotypic space of an organism a more manageable task.

***Estimating the number of experiments needed to infer a complete microbial network***

Though highly speculative, it is interesting to estimate the number of experiments needed to infer a complete network of transcription interactions based on expression data. We can arrive at this number by extrapolating the sensitivity obtained with the CLR algorithm. Assuming that the each experiment processed by the CLR algorithm will reveal an equal fraction of all interactions, q, we can estimate the probability, p_N_, of finding an interaction after N experiments: p_N_ = 1 – (1-q)^N^. The sensitivity of the algorithm is equal to p_N_. At 80% precision with 60 experiments, the CLR algorithm achieves a sensitivity of: p_60_ = 4.5%. Thus we have: p_60_ = 1 – (1-q)^60^ giving q = 0.000769. By extrapolating using this value of q, we can project the number of experiments needed for any sensitivity (p_N_) as N = 60 log(1-p_N_)/log(1- p_60_). For of p_N_ = 70%, this gives: N = 1565 experiments. Similarly, for sensitivities of 80%, 90% and 95% (at 80% precision), we estimate that 2093, 2994 and 3895 experiments would be required. At 60% precision, these numbers become 1108, 1482, 2120, and 2758 experiments at 70%, 80%, 90%, and 95% sensitivity.

We acknowledge that these projections are speculative and likely an underestimate. They assume that (1) all experiments are equally informative and provide as much information as the best experiments in our dataset, and (2) each experiment will elucidate an independent set of interactions. Such an optimal selection is not necessarily possible in a prospectively designed set of experiments. It might also be more accurate to assume that the information value of experiments will diminish as more experiments are performed, because more informative conditions will be selected first. However, the rate at which the information content of the experiments declines (if at all) is unknown. Also, we do not know the relative information value of the experiments in our existing data set, so it would be unrealistic to attempt to estimate this rate from the existing experiments. Thus, we cannot extrapolate using this more sophisticated model. In addition, all transcription factor/target interactions are not likely to be identifiable from expression data alone (see below).

***Limitations of network inference using microarray data***

Although most microbial transcription factors exhibit some modulation of transcription, there may be regulators that are expressed constitutively without any condition-dependent regulation. Modulation of such factors could occur only post-transcriptionally. The targets of such regulators cannot be detected by any algorithm that relies on microarray expression profiles alone, unless they are artificially modulated [31]. Our analysis of existing regulatory pathways suggest that such cases of constitutive expression in microbes are rare; there is nearly always some transcriptional regulation, often through direct or indirect feedback loops or at the level of sigma factor modulation. Nevertheless, detecting the targets of such constitutively transcribed regulators will require an alternative approach based on mass spectrometry, chromatin immunoprecipitation, or synthetic modulation of the regulators. Additionally, current limitations of microarray profiling technology such as the lack of sensitivity for low-expressed genes may limit network inference on this subset of genes, unless the array technology is improved [32].

With all statistical approaches there is a difficult task of differentiating between correlation and causation. Some network inference methods aim to identify causation from the expression data itself by placing assumptions on the network structure. The alternative and best means we have to differentiate between direct regulation and correlated expression is to constrain the network using prior knowledge of transcription factor identity. By allowing only transcription factors to be regulators in the network, we can remove a large number of interactions that are not direct physical links between a transcription factor and its target gene. This prior information also provides direction to all edges except those between two transcription factors.

This constraint, used for all of the algorithms we tested, relies on accurate annotation of transcription factors to determine what genes to define as regulators in the network. Transcription factor identification by sequence analysis is a mature field of bioinformatics. Existing algorithms generate these lists with high sensitivity, but any further improvements in the precision of the list would potentially improve the inferred transcriptional regulatory map as well.

**Scale-free properties of the inferred networks**

Previous studies into global properties of regulatory networks in *E. coli* and *Saccharomyces cerevisiae* have shown a scale-free distribution of out-degree connectivity characterized by a large number of scantly connected nodes and a few highly connected nodes termed hubs [33]. A scale-free distribution was also found in the network inferred by ARACNe using human B cell data [4]. A concern with the background distribution model used by CLR is that it may bias the out-degree of each gene by permitting too many targets for the low-degree nodes and pruning too many targets for the hubs. However, the distribution of out-degree for transcription factors in the CLR inferred network is in fact scale-free (Figure S8). Although the network’s hubs are not as large as some of the RegulonDB hubs, some CLR hubs still have more than fifty targets. The CLR network actually fits a scale-free distribution better than RegulonDB, as the largest hubs in RegulonDB are outliers in the scale-free distribution. All of the other algorithms tested in this study also infer scale-free networks, but all infer smaller hubs on average than the CLR algorithm. These results suggest that if there is any bias in the networks inferred by CLR due to its background distribution model, the bias does not cause a deviation from a scale-free network and imparts less bias on the maximum inferred out-degree than any other tested algorithm.

**Combinatorial Regulation in the CLR Regulatory Map**

As compendiums grow with the reduced cost of microarrays, network inference algorithms will uncover more of the intricate combinatorial regulatory schemes of cells. Currently, there are 824 cases within the known *E. coli* regulatory network, where a gene is regulated by 2-10 transcription factors [5,34]. By adding our new interactions whose precision is 80% or greater, our study increases the number of combinatorially regulated genes by nearly 50.

A simple way to classify combinatorial interactions is to discretize the expression values of each gene and transcription factor into two Boolean states (Figure S9a). The set of all states (the “combinatorial state space”) of the transcription factors and their target under study form a truth table that determines the approximate combinatorial logic function of the transcription factors. For example, in the case of two transcription factors targeting one gene, if the target gene is highly expressed only when both transcription factors are highly expressed, the regulation would be classified as AND-like; whereas if the target gene is highly expressed when either or both of the regulators are highly expressed, the regulation would be classified as OR-like.

Most of the 67 cases of combinatorial regulation involving 2-3 transcription factors, in which at least one of the regulatory interactions was also predicted by our algorithm (the other edges were known edges in RegulonDB), have insufficient data points in the compendium to allow the determination of the logic gate. The combinatorial regulators perturbed in the compendium are usually expressed in the same operon, and thus show high expression covariance leading to undersampling of the combinatorial state space (Figure S9b). Complex combinatorial regulation at such promoters may not occur under normal environmental conditions, or may not be detectable via RNA expression data alone.

There are, nevertheless, some cases where evidence of combinatorial logic can be observed in the expression data. Two cold-shock proteins, CspA and CspG, are identified by CLR to regulate *ddg*, a gene which encodes an enzyme that incorporates palmitoleate instead of laurate into lipid A when *E. coli* undergo cold-shock or growth at below 12°C [35]. The compendium data suggest that the two regulators operate by AND-like logic (Figure S9c and S9d). CspA and CspG are two of the four cold-shock proteins in a quadruple deletion that results in a cold-temperature dependent growth defect [36]. Our results are in concordance with the current working hypothesis that the cold-shock genes evolved by duplication of the *cspA* family genes whereupon the duplicated genes each acquired a more specific role [36]. Our results suggest that CspA is expressed under a large range of temperature conditions, and its presence is required in addition to the more specifically expressed CspG protein to induce the expression of *ddg*, which can then incorporate palmitoleate into lipid A in place of laurate.

**Supplementary Methods**

**Functional Enrichment**

We obtained gene functional annotations and ontology hierarchies from EcoCyc (annotations were from Gene Ontology Consortium, Enzyme Commission (EC), and other ontologies). We included all ancestors of each term associated with a particular gene. We determined enrichment with a hypergeometric distribution by calculating the p-value of the given number of hits for each term based on the query size, the number of genes in the genome, the number of genes in the query with the given association and the number of genes in the genome sharing that association. To ensure that single-gene hits would not provide enrichment to rare categories, we required at least two genes in a query set to map to the same term.

**Motif Detection**

For motif discovery, we first pruned the network at the 60% confidence threshold. We grouped the target genes of each transcription factor into operons using the known and putative operons in RegulonDB [34]. To attain the statistical significance necessary for sequence alignment, we only included transcription factors regulating at least five operons. We constructed multiple alignments of the promoter regions using the MEME multiple alignment system version 3.5.0 [37]. We obtained the operon, predicted operon, and transcription start site annotation from the RegulonDB website. Having obtained the transcription start site, we took 150 bp upstream of this site to be the promoter. When another gene was found less than 150 bp upstream of the transcription start site, we truncated the promoter length to the end of the preceding gene. A background model was built using tri-nucleotide frequencies from all promoter regions. This model was supplied to MEME as the background model for estimating the likelihood of motif occurrence. MEME was constrained to find any number of repetitions of one motif, occurring on the same strand within each promoter. Motif width and other settings were left to default values.

To further assess the significance of each motif, we shuffled the nucleotides of every promoter 100 times and ran MEME on each shuffled dataset, recording the e-value of the top motif each time. These e-values followed a near-normal distribution, skewed by the difference between the common true motif nucleotide distribution and the background distribution. We fit the distribution of shuffled motif e-values to the normal distribution and approximated the quality of the “true” motif as the z-score of that motif's e-value calculated from this ‘shuffle’ background.

**Verification of detected motifs**

We developed a simple algorithm to compare motifs identified by the above procedure to position weight matrices found in PRODORIC [38]. The nucleotide occurrence probability tables reported by MEME were converted to position weight matrices scored in bits (log2), with values of 4 bits of information or less per position. We compared every possible overlap of 10 to 16 positions between each inferred motif and all known motifs. The similarity between the inferred motif and a known motif was scored as:

$$\sum_{\text{pos}} 2-|\text{MEME}_{\text{motif}_{\text{pos}}}-\text{DB}_{\text{motif}_{\text{pos}}}|$$

This score is highest when the position weights for MEME inferred motifs are identical to a known motif in PRODORIC. This procedure allowed ranking of known motifs with respect to the one recovered in our study.

**Chromatin Immunoprecipitation**

Transcription factors assayed by ChIP-PCR were cloned into TOPO (Invitrogen) IPTG inducible vectors containing an Xpress™ epitope. The plasmid was transformed into *E. coli* strain MG1655 and verified by DNA sequencing.

Transcription factor:DNA complexes were immunoprecipitated using a modification of the protocols of Lin and Grossman [39] and Upstate (http://www.upstate.com/). Six replicates were performed for each transcription factor. Cells were diluted 1:100 from overnight cultures into 50 ml of LB with 0.5% glucose in a 250 ml flask and grown to an OD600 of around 0.5. A 15 ml sample was taken from each flask and 400 μl of 37% formaldehyde was added (final concentration 1%). Protein:DNA constructs were cross-linked for 10 minutes at room temperature followed by two washes in ice-cold PBS.

Cells were lysed by incubating samples at 37°C for 30 minutes in 500 μl of lysis buffer (10 mM Tris, 50 mM NaCl, 10 mM EDTA, 20% sucrose, and 4800 units of freshly added Epicenter Ready-lyse lysozyme), followed by addition of 500 μl of 2X IP buffer (200 mM Tris, 600 mM NaCl, 4% Triton X-100, 1 mM fresh PMSF, and 4 μg/ml RNase Cocktail [Ambion]) and incubation for 10 minutes at 37°C with shaking. Lysates were sonicated 4 x 30 seconds with a Branson sonicator on 20% percent power to shear DNA to an average size of 500bp.

A 100 μl sample of the sheared lysate was removed, crosslinks were reversed, and the sheared DNA was purified by phenol:chloroform extraction and ethanol precipitation to determine starting DNA concentration and to verify the shearing size. This purified sheared DNA also served as a positive control for the qPCR step downstream. Three samples, each containing 25 μg of sheared DNA, were taken from the remaining 900 μl of sheared lysate, and they were diluted 1:10 in dilution buffer (1% Triton X-100, 2 mM EDTA, 150 mM NaCl, 20 mM Tris [pH 8], 1 mM PMSF). Two micrograms of antibody specific to the transcription factor epitope tag (Anti-Xpress™) were added to the first sample. Two micrograms of an unrelated antibody (Anti-Myc) were added to the second sample to serve as a negative control. The third sample contained no antibody and served as an additional negative control. All three samples were rotated at 4°C overnight. The following morning, protein A/G agarose beads were added to the samples containing the transcription factor-antibody complexes. The beads were then washed in increasingly stringent conditions (by increasing and changing salts) to remove factors binding non-specifically to the beads or antibody. Protein:DNA complexes were removed from the beads by addition of 500 μl of fresh elution buffer (1% SDS, 100 mM NaHCO_3_) and rotation at room temperature for 15 minutes. Cross-links were reversed by addition of 10 μl of 5M NaCl and incubation at 65°C overnite. The precipitated DNA was purified by phenol:chloroform extraction and ethanol precipitation.

Enrichment of DNA sequences bound to a particular transcription factor was determined by comparing the cycle (Ct) when each qPCR reaction crosses a threshold in the middle of the exponential amplification phase of the reaction. Reactions were performed using an ABI Prism 7900HT with ABI Sybr Green PCR master mix, 150 nM of each primer, and immunoprecipitated DNA template. The log fold-change in enrichment was calculated as $\text{log}((1+E_{i})^{C_{i}-U_{i}})$, where *E_i_* is the median efficiency of the PCR primers for gene *i*, *C_i_* is the Ct value for the DNA enriched using correct antibody for the transcription factor regulating gene *i*, and *U_i_* is the Ct value for the DNA enriched using unrelated antibody for the transcription factor regulating gene *i*. To make the *C_i_-U_i_* enrichment values comparable across each set of replicates, the values for each set of replicates were scaled by the median enrichment value of that set. Outlier enrichment values for each promoter were removed by Grubbs test. Interactions were declared significant when the log fold-change in enrichment for a given gene was significantly greater, by a t-test (P<=0.01) and a non-parametric rank sum test (P<=0.01), than a set of 66 samples taken from the promoter regions of 11 random genes not regulated or inferred to be regulated by the transcription factor being tested. The interactions for Lrp were also tested in Davis minimal media. Lrp interactions enriched in either media were declared significant.

**qPCR Analysis of fecA Combinatorial Regulation**

Strain MG1655 cells were grown in M9 minimal media supplemented with 0.1% casamino acids. Sixteen combinations of sodium citrate (0 mM, 0.25 mM, 0.5 mM, or 0.75 mM) and sodium pyruvate (0%, 0.1%, 0.2%, 0.4%) were added, representing all possible combinations of the three concentrations tested for each chemical. Three to six replicate cultures were grown for each pyruvate/citrate combination. Cells were grown at 37°C to a density of 10^8^ cells/ml as measured by absorbance at 600 nm. Two ml samples of each replicate culture were stabilized in 4 ml of Qiagen RNAprotect reagent. RNA was prepared using Qiagen RNeasy kits. Reverse transcription of 1.5 μg total RNA was performed with 10 units/μL Superscript III Reverse Transcriptase (Invitrogen) using 2.5 mM random hexamers in a total volume of 20 μL, according to the manufacturer’s instructions.

Quantitative PCR primers for the experimental *fecA* transcript, positive control *aceE* transcript, and the normalization transcripts *rrlG* and *rrnA* were designed using Primer Express Software v2.0 (Applied Biosystems). Primer specificity was confirmed with gel electrophoresis. PCR reactions were prepared using 2 μL cDNA in a total volume of 14 μL containing 300 nM of each primer and 7 μL ABI Sybr Green Master Mix. Triplicate PCR reactions were performed and averaged for each of the biological replicates. Reactions were run in an ABI 7900HT.

Crossing-point threshold (Ct) and real-time fluorescence data were obtained using the ABI Prism Sequence Detection Software v2.0. Default software parameters were used except for adjustments made to the pre-exponential phase baseline used to calculate Ct for the higher abundance RNAs. Expression levels were obtained from Ct values as previously described [40].

**Supplementary Figures**

**Figure S5. Examples of typical co-variation in expression between a transcription factor and its target gene.**

(A) Two key players in the SOS response, RecA and its regulatory protein LexA, are both highly expressed in the presence of a DNA damaging agent regardless of the laboratory or experimenter running the microarray. The colors for each point in (A) and (B) correspond to the experimenter. For data from other laboratories, the first author of the associated publication was used. For data from our laboratories, the experimenter name is italicized.

(B) Two genes involved in arabinose metabolism, *araA* and its regulator AraC, behave as a switch that is ON when arabinose is present in the media and OFF otherwise.

**Figure S6. Lrp expression in the compendium**

(A) Confounding experiments and uneven sampling can lead to false correlations (inset) that network inference algorithms aim to eliminate. Addition of the correct condition (in this case, expression profiles on minimal media) clears the relationship.

(B) The relationship between the Lrp transcription factor and its known target *serA* becomes more pronounced upon addition of the minimal media expression profiles in which both genes are highly active.

**Figure S7. Algorithm modifications**

(A) Comparison of multivariate methods. Linear regression networks allow cycles, which provided a performance boost, compared with Bayesian networks. A regression network computed using RMA data only does not perform as well as a network computed from the consensus (average) of four normalization methods (RMA, gcRMA, dChip, and MAS5.0). Random networks represent random guessing of interactions.

(B) The relevance networks algorithm was applied by computing pairwise similarity using mutual information, normalized mutual information, correlation, and by assigning to each gene in an operon the maximum correlation of all genes in the operon. Using correlation as a scoring metric produced the top performing relevance network algorithm.

(C) The original ARACNe algorithm and modified versions of the algorithm were applied. The best performing version of ARACNe employed a probabilistic Data Processing Inequality to prune interactions combined with a spline-based mutual information estimator. Using probabilistic ARACNe, we computed a network using RMA data only, and a network computed from the consensus (average) of four normalization methods. The original ARACNe algorithm uses a discrete mutual information estimator and the standard DPI pruning approach. The spline ARACNe is the same as the original ARACNe, but employs a spline-based mutual information estimator.

(D) The CLR algorithm was applied by computing pairwise similarity using mutual information, normalized mutual information, correlation, and by assigning to each gene in an operon the median or maximum correlation of all genes in the operon. CLR performs better with mutual information than correlation. Prior knowledge of operons improves performance when it is available. The Roulston method is not the CLR algorithm, but is provided for comparison. It computes a background correction using an analytical procedure [11].

**Figure S8. Distribution of out-degree for networks inferred by different algorithms**

All tested algorithms infer networks with an out-degree that is approximately scale-free. The linear regression and CLR methods produced networks with the largest hubs.

**Figure S9. Combinatorial regulation observed in the *E. coli* compendium.**

(A) Combinatorial regulations can be classified by discretizing the expression levels of the relevant transcription factors and genes into on (T) and off (F) states. In this example, two transcription factors, (TF1) and (TF2), regulate their target gene with AND-like logic.

(B) Many of the cases where one gene is regulated by multiple transcription factors involve two transcription factors in the same operon, which eliminates the ability to see either transcription factor expressed independently of the other. This case is difficult to classify as combinatorial regulation.

(C) Novel interactions were predicted between CspA and CspG, two transcription factors involved in cold shock, and the target gene *ddg*, whose expression values are represented as color changes.

(D) The expression profiles for the three cold shock genes strongly suggest an AND-like regulation, but there are no data for the case of high *cspG* expression with low *cspA* expression, thus preventing the conclusive determination of this promoter’s combinatorial logic program.

**Supplementary Tables**

**Table S5. Functional categories with >= 3 unconnected transcription factors at 60% precision**

| **Functional category*** | **Transcription factors** | **Count** |
| --- | --- | --- |
| primary metabolism | ada, asnC, cysB, metJ, metR, mlc, prpR, uidA | 8 |
| prophage genes and phage related functions | appY, dicA, dicC, pspF, yagI, ydaS, yfjR | 7 |
| regulation of cellular metabolism | nagC, ycfQ, ydhB, yeaM, yjhU, ynfL, yphH | 7 |
| energy metabolism, carbon | fhlA, glpR, gntR, hyfR, narP, torR | 6 |
| regulation of transcription, DNA-dependent | ycfQ, ydhB, yeaM, yjhU, ynfL, yphH | 6 |
| regulation of nucleobase, nucleoside, nucleotide and nucleic acid metabolism | ycfQ, ydhB, yeaM, yjhU, ynfL, yphH | 6 |
| cellular biosynthesis | asnC, cysB, metJ, metR, mlc, modE | 6 |
| biosynthesis | asnC, cysB, metJ, metR, mlc, modE | 6 |
| energy derivation by oxidation of organic compounds | fhlA, glpR, hyfR, narP, torR | 5 |
| carboxylic acid metabolism | asnC, cysB, metJ, metR, prpR | 5 |
| generation of precursor metabolites and energy | fhlA, glpR, hyfR, narP, torR | 5 |
| organic acid metabolism | asnC, cysB, metJ, metR, prpR | 5 |
| Transcription related | arcA, creB, kdpE, ompR | 4 |
| RNA related | arcA, creB, kdpE, ompR | 4 |
| anaerobic respiration | glpR, hyfR, narP, torR | 4 |
| carbon utilization | atoC, caiF, putA, uhpA | 4 |
| cellular respiration | glpR, hyfR, narP, torR | 4 |
| sulfur metabolism | aslB, cysB, metJ, metR | 4 |
| amino acid and derivative metabolism | asnC, cysB, metJ, metR | 4 |
| amino acid biosynthesis | asnC, cysB, metJ, metR | 4 |
| amine biosynthesis | asnC, cysB, metJ, metR | 4 |
| amino acid metabolism | asnC, cysB, metJ, metR | 4 |
| nitrogen compound biosynthesis | asnC, cysB, metJ, metR | 4 |
| amine metabolism | asnC, cysB, metJ, metR | 4 |
| nitrogen compound metabolism | asnC, cysB, metJ, metR | 4 |
| response to stimulus | ada, cspE, rpoH, ycaL | 4 |
| type of regulation | ybhN, yddM, yjjQ | 3 |
| nucleoproteins, basic proteins | hns, hupA, hupB | 3 |
| protein related | hns, hupA, hupB | 3 |
| biosynthesis of building blocks | birA, putA, trpR | 3 |
| response to temperature stimulus | cspE, rpoH, ycaL | 3 |
| response to abiotic stimulus | cspE, rpoH, ycaL | 3 |
| sulfur compound biosynthesis | cysB, metJ, metR | 3 |
| aspartate family amino acid biosynthesis | asnC, metJ, metR | 3 |
| sulfur amino acid biosynthesis | cysB, metJ, metR | 3 |
| aspartate family amino acid metabolism | asnC, metJ, metR | 3 |
| sulfur amino acid metabolism | cysB, metJ, metR | 3 |
| macromolecule metabolism | ada, mlc, uidA | 3 |

* generic / nonspecific GO terms have been removed

**Table S6. Functional categories with >= 15 unconnected genes at 60% precision.**

| **Functional Category*** | **Count** |
| --- | --- |
| nucleobase, nucleoside, nucleotide and nucleic acid metabolism | 191 |
| transport | 186 |
| biopolymer metabolism | 171 |
| biosynthesis | 169 |
| cellular biosynthesis | 168 |
| energy metabolism, carbon | 135 |
| energy derivation by oxidation of organic compounds | 135 |
| generation of precursor metabolites and energy | 135 |
| organic acid metabolism | 135 |
| carboxylic acid metabolism | 133 |
| biosynthesis of building blocks | 113 |
| extrachromosomal | 109 |
| carbon utilization | 107 |
| cellular respiration | 100 |
| prophage genes and phage related functions | 99 |
| nitrogen compound metabolism | 96 |
| amine metabolism | 94 |
| amino acid and derivative metabolism | 90 |
| biosynthesis of macromolecules (cellular constituents) | 86 |
| central intermediary metabolism | 85 |
| Channel-type Transporters | 79 |
| carbohydrate metabolism | 76 |
| amino acid metabolism | 76 |
| Pyrophosphate Bond (ATP; GTP; P2) Hydrolysis-driven Active Transporters | 75 |
| catabolism | 74 |
| cellular macromolecule metabolism | 69 |
| The ATP-binding Cassette (ABC) Superfamily + ABC-type Uptake Permeases | 68 |
| anaerobic respiration | 66 |
| carbon compounds | 65 |
| Porters (Uni-, Sym- and Antiporters) | 64 |
| Electrochemical potential driven transporters | 64 |
| RNA metabolism | 64 |
| translation | 59 |
| biopolymer modification | 55 |
| DNA metabolism | 55 |
| macromolecule catabolism | 55 |
| cofactor metabolism | 52 |
| amine biosynthesis | 52 |
| nitrogen compound biosynthesis | 52 |
| cellular protein metabolism | 49 |
| protein metabolism | 49 |
| response to stimulus | 47 |
| cofactor biosynthesis | 46 |
| location of gene products | 45 |
| coenzyme metabolism | 45 |
| RNA modification | 44 |
| cellular carbohydrate metabolism | 44 |
| amino acid biosynthesis | 44 |
| transcriptional activator activity | 44 |
| water-soluble vitamin metabolism | 42 |
| nucleobase, nucleoside and nucleotide interconversion | 42 |
| vitamin metabolism | 42 |
| cellular catabolism | 42 |
| establishment of localization | 41 |
| coenzyme biosynthesis | 39 |
| cell structure | 36 |
| membrane | 34 |
| aerobic respiration | 34 |
| lipid metabolism | 34 |
| cellular lipid metabolism | 34 |
| carbohydrate catabolism | 32 |
| water-soluble vitamin biosynthesis | 31 |
| vitamin biosynthesis | 31 |
| macromolecule biosynthesis | 31 |
| response to stress | 30 |
| carbohydrate biosynthesis | 29 |
| transcriptional repressor activity | 29 |
| DNA replication | 28 |
| aromatic compound metabolism | 27 |
| ABC superfamily, membrane component | 26 |
| lipopolysaccharide | 26 |
| polysaccharide metabolism | 26 |
| ABC superfamily ATP binding cytoplasmic component | 25 |
| outer membrane (sensu Gram-negative Bacteria) | 25 |
| murein (peptidoglycan) | 24 |
| amines | 24 |
| tRNA modification | 24 |
| tRNA metabolism | 24 |
| tRNA aminoacylation for protein translation | 24 |
| amino acid activation | 24 |
| inner membrane | 23 |
| response to abiotic stimulus | 23 |
| cytokinesis | 23 |
| cell division | 23 |
| cellular macromolecule catabolism | 23 |
| regulation of transcription, DNA-dependent | 22 |
| regulation of nucleobase, nucleoside, nucleotide and nucleic acid metabolism | 22 |
| biopolymer catabolism | 22 |
| DNA-dependent DNA replication | 21 |
| aromatic compound biosynthesis | 21 |
| nucleotide metabolism | 20 |
| main pathways of carbohydrate metabolism | 20 |
| cellular polysaccharide metabolism | 20 |
| nucleotide biosynthesis | 20 |
| polysaccharide biosynthesis | 20 |
| biopolymer biosynthesis | 20 |
| cytoplasm | 19 |
| fatty acid oxidation | 19 |
| fatty acid metabolism | 19 |
| protein folding | 19 |
| cellular_component | 19 |
| phosphorous metabolism | 18 |
| heterocycle metabolism | 18 |
| sulfur metabolism | 18 |
| alcohol metabolism | 18 |
| intracellular non-membrane-bound organelle | 18 |
| intracellular organelle | 18 |
| non-membrane-bound organelle | 18 |
| organelle | 18 |
| DNA repair | 17 |
| response to DNA damage stimulus | 17 |
| response to endogenous stimulus | 17 |
| oxidoreduction coenzyme metabolism | 17 |
| glycoprotein metabolism | 17 |
| amino acid derivative metabolism | 16 |
| rRNA metabolism | 16 |
| proteolysis | 16 |
| glycopeptide catabolism | 16 |
| glycoprotein catabolism | 16 |
| proteolysis during cellular protein catabolism | 16 |
| cellular protein catabolism | 16 |
| protein catabolism | 16 |
| protection | 15 |
| fermentation | 15 |
| phospholipid metabolism | 15 |
| membrane lipid metabolism | 15 |
| pteridine and derivative metabolism | 15 |
| pteridine and derivative biosynthesis | 15 |
| carboxylic acid biosynthesis | 15 |
| organic acid biosynthesis | 15 |
| phospholipid biosynthesis | 15 |
| membrane lipid biosynthesis | 15 |
| lipid biosynthesis | 15 |
| transporter activity | 15 |

* generic / nonspecific GO terms have been removed

**Protocol S1 References**

1. Walker GC (1996) The SOS Response of *Escherichia coli*. In: Neidhardt FC, Curtiss R, editors. Escherichia coli and Salmonella: cellular and molecular biology. 2nd ed. Washington, D.C.: ASM Press.

2. Margolin AA, Nemenman I, Basso K, Wiggins C, Stolovitzky G, et al. (2006) ARACNE: an algorithm for the reconstruction of gene regulatory networks in a mammalian cellular context. BMC Bioinformatics 7 Suppl 1: S7.

3. Hartemink AJ (2005) Reverse engineering gene regulatory networks. Nat Biotechnol 23: 554-555.

4. Basso K, Margolin AA, Stolovitzky G, Klein U, Dalla-Favera R, et al. (2005) Reverse engineering of regulatory networks in human B cells. Nat Genet 37: 382-390.

5. Shen-Orr SS, Milo R, Mangan S, Alon U (2002) Network motifs in the transcriptional regulation network of Escherichia coli. Nat Genet 31: 64-68.

6. Hartemink AJ, Gifford DK, Jaakkola TS, Young RA (2002) Combining location and expression data for principled discovery of genetic regulatory network models. Pac Symp Biocomput: 437-449.

7. Murphy K (2001) The Bayes Net Toolbox for Matlab. Computing Science and Statistics 33.

8. Friedman N, Linial M, Nachman I, Pe'er D (2000) Using Bayesian networks to analyze expression data. J Comput Biol 7: 601-620.

9. Butte AJ, Tamayo P, Slonim D, Golub TR, Kohane IS (2000) Discovering functional relationships between RNA expression and chemotherapeutic susceptibility using relevance networks. Proc Natl Acad Sci U S A 97: 12182-12186.

10. Butte AJ, Kohane IS (2000) Mutual information relevance networks: functional genomic clustering using pairwise entropy measurements. Pac Symp Biocomput: 418-429.

11. Roulston M (1997) Significance testing of information theoretic functionals. Physica D Volume 110, Number 1: 62-66 (65).

12. Qian J, Lin J, Luscombe NM, Yu H, Gerstein M (2003) Prediction of regulatory networks: genome-wide identification of transcription factor targets from gene expression data. Bioinformatics 19: 1917-1926.

13. Liang S, Fuhrman S, Somogyi R (1998) Reveal, a general reverse engineering algorithm for inference of genetic network architectures. Pac Symp Biocomput: 18-29.

14. de la Fuente A, Brazhnik P, Mendes P (2002) Linking the genes: inferring quantitative gene networks from microarray data. Trends Genet 18: 395-398.

15. Ideker T, Thorsson V, Ranish JA, Christmas R, Buhler J, et al. (2001) Integrated genomic and proteomic analyses of a systematically perturbed metabolic network. Science 292: 929-934.

16. Wagner A (2001) How to reconstruct a large genetic network from n gene perturbations in fewer than n(2) easy steps. Bioinformatics 17: 1183-1197.

17. Tringe SG, Wagner A, Ruby SW (2004) Enriching for direct regulatory targets in perturbed gene-expression profiles. Genome Biol 5: R29.

18. Ideker TE, Thorsson V, Karp RM (2000) Discovery of regulatory interactions through perturbation: inference and experimental design. Pac Symp Biocomput: 305-316.

19. D'Haeseleer P, Liang S, Somogyi R (2000) Genetic network inference: from co-expression clustering to reverse engineering. Bioinformatics 16: 707-726.

20. Bonneau R, Reiss DJ, Shannon P, Facciotti M, Hood L, et al. (2006) The Inferelator: an algorithm for learning parsimonious regulatory networks from systems-biology data sets de novo. Genome Biol 7: R36.

21. D'Haeseleer P, Wen X, Fuhrman S, Somogyi R (1999) Linear modeling of mRNA expression levels during CNS development and injury. Pac Symp Biocomput: 41-52.

22. Bansal M, Gatta GD, di Bernardo D (2006) Inference of gene regulatory networks and compound mode of action from time course gene expression profiles. Bioinformatics 22: 815-822.

23. Kaeberlein T, Lewis K, Epstein SS (2002) Isolating "uncultivable" microorganisms in pure culture in a simulated natural environment. Science 296: 1127-1129.

24. Mashburn LM, Whiteley M (2005) Membrane vesicles traffic signals and facilitate group activities in a prokaryote. Nature 437: 422-425.

25. Vlamakis HC, Kolter R (2005) Thieves, assassins and spies of the microbial world. Nat Cell Biol 7: 933-934.

26. Xavier KB, Bassler BL (2005) Interference with AI-2-mediated bacterial cell-cell communication. Nature 437: 750-753.

27. Box GEP, Hunter WG, Hunter JS (1978) Statistics for experimenters: an introduction to design, data analysis, and model building. New York: Wiley. xviii, 653 p.

28. Fisher RA (1966) The design of experiments. New York: Hafner Pub. Co. xv, 248 p.

29. Kerr MK, Churchill GA (2001) Experimental design for gene expression microarrays. Biostatistics 2: 183-201.

30. Scholtens D, Miron A, Merchant FM, Miller A, Miron PL, et al. (2004) Analyzing factorial designed microarray experiments. Journal of Multivariate Analysis 90: 19-43.

31. Gardner TS, Faith JJ (2005) Reverse-engineering transcription control networks. Physics of Life Reviews 2: 65-88.

32. Draghici S, Khatri P, Eklund AC, Szallasi Z (2006) Reliability and reproducibility issues in DNA microarray measurements. Trends Genet 22: 101-109.

33. Balazsi G, Barabasi AL, Oltvai ZN (2005) Topological units of environmental signal processing in the transcriptional regulatory network of Escherichia coli. Proc Natl Acad Sci U S A 102: 7841-7846.

34. Salgado H, Gama-Castro S, Peralta-Gil M, Diaz-Peredo E, Sanchez-Solano F, et al. (2006) RegulonDB (version 5.0): Escherichia coli K-12 transcriptional regulatory network, operon organization, and growth conditions. Nucleic Acids Res 34: D394-397.

35. Carty SM, Sreekumar KR, Raetz CR (1999) Effect of cold shock on lipid A biosynthesis in Escherichia coli. Induction At 12 degrees C of an acyltransferase specific for palmitoleoyl-acyl carrier protein. J Biol Chem 274: 9677-9685.

36. Xia B, Ke H, Inouye M (2001) Acquirement of cold sensitivity by quadruple deletion of the cspA family and its suppression by PNPase S1 domain in Escherichia coli. Mol Microbiol 40: 179-188.

37. Bailey TL, Elkan C (1994) Fitting a mixture model by expectation maximization to discover motifs in biopolymers. Proc Int Conf Intell Syst Mol Biol 2: 28-36.

38. Munch R, Hiller K, Barg H, Heldt D, Linz S, et al. (2003) PRODORIC: prokaryotic database of gene regulation. Nucleic Acids Res 31: 266-269.

39. Lin DC, Grossman AD (1998) Identification and characterization of a bacterial chromosome partitioning site. Cell 92: 675-685.

40. Gardner TS, di Bernardo D, Lorenz D, Collins JJ (2003) Inferring genetic networks and identifying compound mode of action via expression profiling. Science 301: 102-105.
