# Supplementary material for: Large-Scale Mapping and Validation of Escherichia coli Transcriptional Regulation from a Compendium of Expression Profiles
Source: PLoS Biol. 2007 Jan 9;5(1):e8. doi: 10.1371/journal.pbio.0050008 (PMC1764438; doi:10.1371/journal.pbio.0050008)
Supplement: Supplementary file 6 [file pbio.0050008.st001.doc]

**Table S1. Functional enrichment of YmfN targets.**

| **Functional category** | **Target Genes** | **p-value** |
| --- | --- | --- |
| prophage genes and phage related functions | ymfR, ymfO, ymfM, ymfL, intE | 0.0001 |
| Extrachromosomal | ymfR, ymfO, ymfM, ymfL, intE | 0.0004 |
| DNA repair | recN, dinD | 0.0029 |
| response to DNA damage stimulus | recN, dinD | 0.0029 |
| response to endogenous stimulus | recN, dinD | 0.0029 |
| response to stress | recN, dinD | 0.0081 |
| DNA related | recN, dinD | 0.01 |
| response to stimulus | recN, dinD | 0.0236 |
| DNA metabolism | recN, dinD | 0.0251 |
