# Supplementary material for: Large-Scale Mapping and Validation of Escherichia coli Transcriptional Regulation from a Compendium of Expression Profiles
Source: PLoS Biol. 2007 Jan 9;5(1):e8. doi: 10.1371/journal.pbio.0050008 (PMC1764438; doi:10.1371/journal.pbio.0050008)
Supplement: Supplementary file 7 [file pbio.0050008.st002.doc]

**Table S2. Functional enrichment of YnaE targets.**

| **Functional category** | **Target Genes** | **p-value** |
| --- | --- | --- |
| response to temperature stimulus | cspI, cspG | 0.0004 |
| response to abiotic stimulus | cspI,cspG | 0.002 |
| response to stimulus | cspI,cspG | 0.0106 |
