# Supplementary material for: Large-Scale Mapping and Validation of Escherichia coli Transcriptional Regulation from a Compendium of Expression Profiles
Source: PLoS Biol. 2007 Jan 9;5(1):e8. doi: 10.1371/journal.pbio.0050008 (PMC1764438; doi:10.1371/journal.pbio.0050008)
Supplement: Supplementary file 8 [file pbio.0050008.st003.doc]

**Table S3. z-scores of motifs for transcription factors in the 60% precision network with >= 5 predicted operon targets.**

| **Shaded rows indicate z >= 1.64 (> 0.95 one-sided significance)** | | | | | |
| --- | --- | --- | --- | --- | --- |
| **Regulator** | **# of operons** | **z-score** | **Motif rank**  **(if motif is known)** | **Known motif source** | **Possible confounding regulator (match rank)** |
| alpA_b2624_at | 5 | 3.39 |  |  |  |
| araC_b0064_at | 6 | 1.64 | 1 | DPInteract |  |
| arsR_b3501_at | 16 | 2.78 |  |  |  |
| b0373_s_at | 6 | 5.01 |  |  |  |
| b1422_at | 5 | 0.85 |  |  |  |
| b2531_at | 5 | 4.12 |  |  |  |
| bolA_b0435_at | 5 | 1.14 |  |  |  |
| celD_b1735_at | 8 | 1.45 |  |  |  |
| cspB_b1557_at | 5 | 0.45 |  |  |  |
| cspG_b0990_at | 8 | 2.06 |  |  |  |
| cspI_b1552_at | 6 | 0.27 |  |  |  |
| dnaA_b3702_at | 11 | 0.32 | 33 | DPInteract |  |
| fecI_b4293_at | 7 | 6.21 |  |  | Fur(1) |
| fis_b3261_at | 5 | 2.44 | 3 | DPInteract |  |
| flhC_b1891_at | 19 | 4.05 | 3 | Regulon |  |
| flhD_b1892_at | 18 | 4.80 | 3 | Regulon |  |
| fliA_b1922_at | 20 | 4.89 | 18 | PRODORIC | FlhDC(3) |
| fnr_b1334_at | 9 | 2.28 | 13 | PRODORIC |  |
| frvR_b3897_at | 5 | 0.41 |  |  |  |
| fucR_b2805_at | 7 | -0.36 |  |  |  |
| gcvR_b2479_at | 5 | 1.28 |  |  | GcvA(6) |
| glcC_b2980_at | 9 | 0.62 |  |  |  |
| htgA_b0012_at | 8 | 0.46 |  |  |  |
| hycA_b2725_at | 7 | 2.50 |  |  |  |
| leuO_b0076_at | 8 | 3.47 |  |  |  |
| lexA_b4043_at | 13 | 9.39 | 1 | DPInteract |  |
| lldR_b3604_at | 5 | -0.38 |  |  |  |
| lrp_b0889_at | 27 | 4.63 | 15 | DPInteract | ArgR(1) |
| mhpR_b0346_at | 12 | 5.52 |  |  |  |
| nadR_b4390_at | 6 | 2.17 |  |  |  |
| osmE_b1739_at | 33 | 4.16 |  |  |  |
| phnF_b4102_at | 7 | 3.10 |  |  |  |
| rhaR_b3906_at | 9 | 0.09 | 41 | Regulon | RhaS(3) |
| rhaS_b3905_at | 12 | 3.85 | 7 | DPInteract |  |
| rpoN_b3202_at | 6 | 0.13 | 16 | PRODORIC | RpoH(4) |
| rstA_b1608_at | 5 | 0.48 |  |  |  |
| tdcR_b3119_at | 8 | 0.60 |  |  |  |
| ybaQ_b0483_at | 6 | 1.66 |  |  |  |
| ycjC_b1299_at | 5 | 1.26 |  |  |  |
| ydaK_b1339_at | 7 | 0.18 |  |  |  |
| ydaR_b1356_at | 5 | -0.59 |  |  |  |
| yebK_b1853_at | 11 | -0.81 |  |  |  |
| yfeC_b2398_at | 5 | -0.59 |  |  | CaiF(1) |
| yfeD_b2399_at | 6 | 0.23 |  |  |  |
| ygeK_b2855_at | 8 | 0.35 |  |  |  |
| yheN_b3345_at | 6 | 0.83 |  |  |  |
| yhiE_b3512_at | 20 | 3.30 |  |  | YhiX(13) |
| yhiF_b3507_at | 9 | 3.70 |  |  | YhiX(7) |
| yhiW_b3515_at | 9 | -0.09 |  |  | yhiX(19) |
| yhiX_b3516_at | 16 | 4.21 | 4 | PRODORIC |  |
| yhjB_b3520_at | 10 | 2.85 |  |  | CysB(1) |
| yidF_b3674_at | 9 | 0.49 |  |  |  |
| yidW_b3695_at | 5 | -0.47 |  |  |  |
| yihL_b3872_at | 5 | 0.88 |  |  |  |
| yjaE_b3995_at | 6 | 1.44 |  |  |  |
| yjbK_b4046_at | 10 | 1.34 |  |  |  |
| ymfL_b1147_at | 7 | 0.84 |  |  |  |
| ymfN_b1149_at | 6 | 1.79 |  |  |  |
| ynaE_b1375_s_at | 8 | 5.34 |  |  |  |
| yneJ_b1526_at | 6 | -0.41 |  |  |  |
| yrbA_b3190_at | 10 | 0.19 |  |  |  |
|  |  |  |  |  |  |
| **% significant (z >= 1.64)** | **48.4375** |  |  |  |  |
|  |  |  |  |  |  |
|  |  |  |  |  |  |
|  |  |  |  |  |  |
