# Supplementary material for: Large-Scale Mapping and Validation of Escherichia coli Transcriptional Regulation from a Compendium of Expression Profiles
Source: PLoS Biol. 2007 Jan 9;5(1):e8. doi: 10.1371/journal.pbio.0050008 (PMC1764438; doi:10.1371/journal.pbio.0050008)
Supplement: Supplementary file 9 [file pbio.0050008.st004.doc]

**Table S4. The clustered microarrays of the *E. coli* compendium.**

*TP* in the Reference column indicates expression profiles contributed by this paper.

| **Cluster** | **Chip name** | **Experiment description** | **Reference** |
| --- | --- | --- | --- |
| 1 | dinI___U_N0025_r1 | dinI upregulation, amp 50ug/ml, 0.0125% arabinose, 0.025 ug/ml norfloxacin OD ~0.3 | *TP* |
| 1 | luc___U_N0000_r1 | luc upregulation, 0.000 ug/ml norfloxacin | *TP* |
| 1 | luc___U_N0000_r2 | luc upregulation, 0.000 ug/ml norfloxacin | *TP* |
| 1 | luc___U_N0000_r3 | luc upregulation, 0.000 ug/ml norfloxacin | *TP* |
| 2 | dinI___U_N0025_r2 | dinI upregulation, amp 50 ug/ml, 0.0125% arabinose, 0.025 ug/ml norfloxacin OD ~0.3 | *TP* |
| 2 | dinI___U_N0025_r3 | dinI upregulation, amp 50 ug/ml, 0.0125% arabinose, 0.025 ug/ml norfloxacin OD ~0.3 | *TP* |
| 2 | dinP___U_N0025_r1 | dinP upregulation, amp 50 ug/ml, 0.0125% arabinose, 0.025 ug/ml norfloxacin OD ~0.3 | *TP* |
| 2 | dinP___U_N0025_r2 | dinP upregulation, amp 50 ug/ml, 0.0125% arabinose, 0.025 ug/ml norfloxacin OD ~0.3 | *TP* |
| 2 | dinP___U_N0025_r3 | dinP upregulation, amp 50 ug/ml, 0.0125% arabinose, 0.025 ug/ml norfloxacin OD ~0.3 | *TP* |
| 2 | lexA___U_N0025_r1 | lexA upregulation, amp 50 ug/ml, 0.0125% arabinose, 0.025 ug/ml norfloxacin OD ~0.3 | *TP* |
| 2 | lexA___U_N0025_r2 | lexA upregulation, amp 50 ug/ml, 0.0125% arabinose, 0.025 ug/ml norfloxacin OD ~0.3 | *TP* |
| 2 | lexA___U_N0025_r3 | lexA upregulation, amp 50 ug/ml, 0.0125% arabinose, 0.025 ug/ml norfloxacin OD ~0.3 | *TP* |
| 2 | lon___U_N0025_r1 | lon upregulation, amp 50 ug/ml, 0.0125% arabinose, 0.025 ug/ml norfloxacin OD ~0.3 | *TP* |
| 2 | lon___U_N0025_r2 | Lon upregulation, amp 50 ug/ml, 0.0125% arabinose, 0.025 ug/ml norfloxacin OD ~0.3 | *TP* |
| 2 | lon___U_N0025_r3 | lon upregulation, amp 50 ug/ml, 0.0125% arabinose, 0.025 ug/ml norfloxacin OD ~0.3 | *TP* |
| 2 | luc___U_N0025_r1 | luc upregulation, amp 50 ug/ml, 0.0125% arabinose, 0.025 ug/ml norfloxacin OD ~0.3 | *TP* |
| 2 | luc___U_N0025_r2 | luc upregulation, amp 50 ug/ml, 0.0125% arabinose, 0.025 ug/ml norfloxacin OD ~0.3 | *TP* |
| 2 | luc___U_N0025_r3 | luc upregulation, amp 50 ug/ml, 0.0125% arabinose, 0.025 ug/ml norfloxacin OD ~0.3 | *TP* |
| 2 | recA___U_N0025_r1 | recA upregulation, amp 50 ug/ml, 0.0125% arabinose, 0.025 ug/ml norfloxacin OD ~0.3 | *TP* |
| 2 | recA___U_N0025_r2 | recA upregulation, amp 50 ug/ml, 0.0125% arabinose, 0.025 ug/ml norfloxacin OD ~0.3 | *TP* |
| 2 | recA___U_N0025_r3 | recA upregulation, amp 50 ug/ml, 0.0125% arabinose, 0.025 ug/ml norfloxacin OD ~0.3 | *TP* |
| 2 | ruvA___U_N0025_r1 | ruvA upregulation, amp 50 ug/ml, 0.0125% arabinose, 0.025 ug/ml norfloxacin OD ~0.3 | *TP* |
| 2 | ruvA___U_N0025_r2 | ruvA upregulation, amp 50 ug/ml, 0.0125% arabinose, 0.025 ug/ml norfloxacin OD ~0.3 | *TP* |
| 2 | ruvA___U_N0025_r3 | ruvA upregulation, amp 50 ug/ml, 0.0125% arabinose, 0.025 ug/ml norfloxacin OD ~0.3 | *TP* |
| 2 | sulA___U_N0025_r1 | sulA upregulation, amp 50 ug/ml, 0.0125% arabinose, 0.025 ug/ml norfloxacin OD ~0.3 | *TP* |
| 2 | sulA___U_N0025_r2 | sulA upregulation, amp 50 ug/ml, 0.0125% arabinose, 0.025 ug/ml norfloxacin OD ~0.3 | *TP* |
| 2 | sulA___U_N0025_r3 | sulA upregulation, amp 50 ug/ml, 0.0125% arabinose, 0.025 ug/ml norfloxacin OD ~0.3 | *TP* |
| 2 | umuD___U_N0025_r1 | umuD upregulation, amp 50 ug/ml, 0.0125% arabinose, 0.025 ug/ml norfloxacin OD ~0.3 | *TP* |
| 2 | umuD___U_N0025_r2 | umuD upregulation, amp 50 ug/ml, 0.0125% arabinose, 0.025 ug/ml norfloxacin OD ~0.3 | *TP* |
| 2 | umuD___U_N0025_r3 | umuD upregulation, amp 50 ug/ml, 0.0125% arabinose, 0.025 ug/ml norfloxacin OD ~0.3 | *TP* |
| 2 | uvrA___U_N0025_r1 | uvrA upregulation, amp 50 ug/ml, 0.0125% arabinose, 0.025 ug/ml norfloxacin OD ~0.3 | *TP* |
| 2 | uvrA___U_N0025_r2 | uvrA upregulation, amp 50 ug/ml, 0.0125% arabinose, 0.025 ug/ml norfloxacin OD ~0.3 | *TP* |
| 2 | uvrA___U_N0025_r3 | uvrA upregulation, amp 50 ug/ml, 0.0125% arabinose, 0.025 ug/ml norfloxacin OD ~0.3 | *TP* |
| 3 | dnaA__U_N0075_r1 | dnaA upregulation, 0.075 ug/ml norfloxacin | *TP* |
| 3 | gyrA__U_N0075_r1 | gyrA upregulation, 0.075 ug/mL norfloxacin | *TP* |
| 3 | gyrI__U_N0075_r2 | gyrI upregulation, 0.075 ug/mL norfloxacin | *TP* |
| 3 | minD__U_N0075_r1 | minD upregulation, 0.075 ug/mL norfloxacin | *TP* |
| 3 | murI__U_N0075_r1 | murI upregulation, 0.075 ug/mL norfloxacin | *TP* |
| 3 | rstB__U_N0075_r1 | rstB upregulation, 0.075 ug/mL norfloxacin | *TP* |
| 3 | uspA__U_N0075_r1 | uspA upregulation, 0.075 ug/mL norfloxacin | *TP* |
| 4 | dnaA__U_N0075_r2 | dnaA upregulation, 0.075 ug/ml norfloxacin | *TP* |
| 4 | gyrI__U_N0075_r1 | gyrI upregulation, 0.075 ug/mL norfloxacin | *TP* |
| 4 | menB__U_N0075_r1 | menB upregulation, 0.075 ug/mL norfloxacin | *TP* |
| 5 | dnaN__U_N0075_r1 | dnaN upregulation, 0.075 ug/ml norfloxacin | *TP* |
| 5 | dnaT__U_N0075_r1 | dnaT upregulation, 0.075 ug/ml norfloxacin | *TP* |
| 5 | sbcB__U_N0075_r1 | sbcB upregulation, 0.075 ug/mL norfloxacin | *TP* |
| 6 | dnaN__U_N0075_r2 | dnaN upregulation, 0.075 ug/ml norfloxacin | *TP* |
| 6 | dnaT__U_N0075_r2 | dnaT upregulation, 0.075 ug/ml norfloxacin | *TP* |
| 6 | hscA__U_N0075_r2 | hscA upregulation, 0.075 ug/mL norfloxacin | *TP* |
| 6 | minE__U_N0075_r2 | minE upregulation, 0.075 ug/mL norfloxacin | *TP* |
| 6 | murI__U_N0075_r2 | murI upregulation, 0.075 ug/mL norfloxacin | *TP* |
| 6 | sbcB__U_N0075_r2 | sbcB upregulation, 0.075 ug/mL norfloxacin | *TP* |
| 7 | emrR__U_N0075_r1 | emrR upregulation, 0.075 ug/ml norfloxacin | *TP* |
| 7 | holD__U_N0075_r1 | holD upregulation, 0.075 ug/mL norfloxacin | *TP* |
| 7 | hscA__U_N0075_r1 | hscA upregulation, 0.075 ug/mL norfloxacin | *TP* |
| 7 | IHF___U_N0075_r1 | IHF upregulation, 0.075 ug/mL norfloxacin | *TP* |
| 7 | minE__U_N0075_r1 | minE upregulation, 0.075 ug/mL norfloxacin | *TP* |
| 7 | nrdA__U_N0075_r1 | nrdA upregulation, 0.075 ug/mL norfloxacin | *TP* |
| 7 | nrdB__U_N0075_r1 | nrdB upregulation, 0.075 ug/mL norfloxacin | *TP* |
| 8 | emrR__U_N0075_r3 | emrR upregulation, 0.075 ug/ml norfloxacin | *TP* |
| 8 | hlpA__U_N0075_r2 | hlpA upregulation, 0.075 ug/mL norfloxacin | *TP* |
| 8 | holD__U_N0075_r2 | holD upregulation, 0.075 ug/mL norfloxacin | *TP* |
| 8 | hscA__U_N0075_r3 | hscA upregulation, 0.075 ug/mL norfloxacin | *TP* |
| 8 | IHF___U_N0075_r3 | IHF upregulation, 0.075 ug/mL norfloxacin | *TP* |
| 8 | nrdA__U_N0075_r3 | nrdA upregulation, 0.075 ug/mL norfloxacin | *TP* |
| 8 | nrdB__U_N0075_r3 | nrdB upregulation, 0.075 ug/mL norfloxacin | *TP* |
| 8 | ruvC__U_N0075_r1 | ruvC upregulation, 0.075 ug/mL norfloxacin | *TP* |
| 9 | folA__U_N0075_r2 | folA upregulation, 0.075 ug/ml norfloxacin | *TP* |
| 9 | menC__U_N0075_r2 | menC upregulation, 0.075 ug/mL norfloxacin | *TP* |
| 10 | galF__U_N0075_r1 | galF upregulation, 0.075 ug/ml norfloxacin | *TP* |
| 10 | hlpA__U_N0075_r1 | hlpA upregulation, 0.075 ug/mL norfloxacin | *TP* |
| 10 | menC__U_N0075_r3 | menC upregulation, 0.075 ug/mL norfloxacin | *TP* |
| 11 | galF__U_N0075_r2 | galF upregulation, 0.075 ug/ml norfloxacin | *TP* |
| 11 | nupC__U_N0075_r2 | nupC upregulation, 0.075 ug/mL norfloxacin | *TP* |
| 12 | gcvR__U_N0075_r1 | gcvR upregulation, 0.075 ug/mL norfloxacin | *TP* |
| 12 | gyrI__U_N0075_r3 | gyrI upregulation, 0.075 ug/mL norfloxacin | *TP* |
| 12 | holD__U_N0075_r3 | holD upregulation, 0.075 ug/mL norfloxacin | *TP* |
| 12 | minD__U_N0075_r3 | minD upregulation, 0.075 ug/mL norfloxacin | *TP* |
| 12 | murI__U_N0075_r3 | murI upregulation, 0.075 ug/mL norfloxacin | *TP* |
| 12 | rimI__U_N0075_r1 | rimI upregulation, 0.075 ug/mL norfloxacin | *TP* |
| 12 | rimI__U_N0075_r3 | rimI upregulation, 0.075 ug/mL norfloxacin | *TP* |
| 12 | rstB__U_N0075_r3 | rstB upregulation, 0.075 ug/mL norfloxacin | *TP* |
| 12 | ruvC__U_N0075_r3 | ruvC upregulation, 0.075 ug/mL norfloxacin | *TP* |
| 12 | sbcB__U_N0075_r3 | sbcB upregulation, 0.075 ug/mL norfloxacin | *TP* |
| 13 | menC__U_N0075_r1 | menC upregulation, 0.075 ug/mL norfloxacin | *TP* |
| 13 | minD__U_N0075_r2 | minD upregulation, 0.075 ug/mL norfloxacin | *TP* |
| 14 | yoeB__U_N0075_r1 | yoeB upregulation, 0.075 ug/mL norfloxacin | *TP* |
| 14 | yoeB__U_N0075_r2 | yoeB upregulation, 0.075 ug/mL norfloxacin | *TP* |
| 14 | yoeB__U_N0075_r3 | yoeB upregulation, 0.075 ug/mL norfloxacin | *TP* |
| 15 | nupC__U_N0075_r3 | nupC upregulation, 0.075 ug/mL norfloxacin | *TP* |
| 16 | mazF___U_N0025_r1 | mazF_chpA upregulation, amp 50 ug/ml, 0.0125% arabinose, 0.025 ug/ml norfloxacin OD ~0.3 | *TP* |
| 16 | mazF___U_N0025_r2 | mazF_chpA upregulation, amp 50 ug/ml, 0.0125% arabinose, 0.025 ug/ml norfloxacin OD ~0.3 | *TP* |
| 16 | mazF___U_N0025_r3 | mazF_chpA upregulation, amp 50 ug/ml, 0.0125% arabinose, 0.025 ug/ml norfloxacin OD ~0.3 | *TP* |
| 16 | relE___U_N0025_r1 | relE upregulation, amp 50 ug/ml, 0.0125% arabinose, 0.025 ug/ml norfloxacin OD ~0.3 | *TP* |
| 16 | relE___U_N0025_r2 | relE upregulation, amp 50 ug/ml, 0.0125% arabinose, 0.025 ug/ml norfloxacin OD ~0.3 | *TP* |
| 16 | relE___U_N0025_r3 | relE upregulation, amp 50 ug/ml, 0.0125% arabinose, 0.025 ug/ml norfloxacin OD ~0.3 | *TP* |
| 17 | WT_N0000_r1 | wild-type cells without norfloxacin | *TP* |
| 17 | WT_N0000_r2 | wild-type cells without norfloxacin | *TP* |
| 17 | WT_N0025_r1 | wild-type cells with 0.025 ug/ml norfloxacin | *TP* |
| 17 | WT_N0025_r2 | wild-type cells with 0.025 ug/ml norfloxacin | *TP* |
| 17 | WT_N0050_r1 | wild-type cells with 0.050 ug/ml norfloxacin | *TP* |
| 17 | WT_N0050_r2 | wild-type cells with 0.050 ug/ml norfloxacin | *TP* |
| 18 | WT_N0075_r1 | wild-type cells with 0.075 ug/ml norfloxacin | *TP* |
| 18 | WT_N0075_r2 | wild-type cells with 0.075 ug/ml norfloxacin | *TP* |
| 19 | recA_D_N0000_r1 | recA deletion 0 ug/ml norfloxacin | *TP* |
| 19 | recA_D_N0000_r2 | recA deletion 0 ug/ml norfloxacin | *TP* |
| 19 | recA_D_N0050_r1 | recA deletion 0.05 ug/ml norfloxacin | *TP* |
| 19 | recA_D_N0050_r2 | recA deletion 0.05 ug/ml norfloxacin | *TP* |
| 19 | recA_D_N1000_r1 | recA deletion 1.0 ug/ml norfloxacin | *TP* |
| 19 | recA_D_N1000_r2 | recA deletion 1.0 ug/ml norfloxacin | *TP* |
| 19 | WT_D_N1000_r1 | wild-type 1.0 ug/ml norfloxacin | *TP* |
| 19 | WT_D_N1000_r2 | wild-type 1.0 ug/ml norfloxacin | *TP* |
| 20 | luc2_U_N0025_r1 | luciferase 0.025 ug/ml norfloxacin | *TP* |
| 20 | luc2_U_N0000_r1 | luciferase no drug | *TP* |
| 21 | luc2_U_N0025_r2 | luciferase 0.025 ug/ml norfloxacin | *TP* |
| 21 | luc2_U_N0000_r2 | luciferase no drug | *TP* |
| 22 | T0_N0025_r1 | cells prior to treatment by norfloxacin | *TP* |
| 22 | T0_N0025_r2 | cells prior to treatment by norfloxacin | *TP* |
| 22 | T0_N0025_r3 | cells prior to treatment by norfloxacin | *TP* |
| 23 | T12_N0025_r1 | cells 12 min after treatment by norfloxacin | *TP* |
| 23 | T12_N0025_r2 | cells 12 min after treatment by norfloxacin | *TP* |
| 23 | T12_N0025_r3 | cells 12 min after treatment by norfloxacin | *TP* |
| 23 | T24_N0025_r1 | cells 24 min after treatment by norfloxacin | *TP* |
| 23 | T24_N0025_r2 | cells 24 min after treatment by norfloxacin | *TP* |
| 23 | T24_N0025_r3 | cells 24 min after treatment by norfloxacin | *TP* |
| 23 | T36_N0025_r1 | cells 36 min after treatment by norfloxacin | *TP* |
| 23 | T36_N0025_r2 | cells 36 min after treatment by norfloxacin | *TP* |
| 23 | T36_N0025_r3 | cells 36 min after treatment by norfloxacin | *TP* |
| 23 | T48_N0025_r1 | cells 48 min after treatment by norfloxacin | *TP* |
| 23 | T48_N0025_r2 | cells 48 min after treatment by norfloxacin | *TP* |
| 23 | T48_N0025_r3 | cells 48 min after treatment by norfloxacin | *TP* |
| 23 | T60_N0025_r1 | cells 60 min after treatment by norfloxacin | *TP* |
| 23 | T60_N0025_r2 | cells 60 min after treatment by norfloxacin | *TP* |
| 23 | T60_N0025_r3 | cells 60 min after treatment by norfloxacin | *TP* |
| 24 | T24_N0000_r1 | untreated cells after 24 min | *TP* |
| 24 | T24_N0000_r2 | untreated cells after 24 min | *TP* |
| 24 | T24_N0000_r3 | untreated cells after 24 min | *TP* |
| 24 | T60_N0000_r1 | untreated cells after 60 min | *TP* |
| 24 | T60_N0000_r2 | untreated cells after 60 min | *TP* |
| 24 | T60_N0000_r3 | untreated cells after 60 min | *TP* |
| 25 | ccdB_K12_0_r1 | E.coli K12 with ccdB upregulation 0 minutes after induction | *TP* |
| 25 | lacZ_K12_0_r1 | E.coli K12 with lacZ upregulation 0 minutes after induction | *TP* |
| 26 | ccdB_K12_30_r1 | E.coli K12 with ccdB upregulation 30 minutes after induction | *TP* |
| 26 | ccdB_K12_60_r1 | E.coli K12 with ccdB upregulation 60 minutes after induction | *TP* |
| 26 | lacZ_K12_30_r1 | E.coli K12 with lacZ upregulation 30 minutes after induction | *TP* |
| 26 | lacZ_K12_60_r1 | E.coli K12 with lacZ upregulation 60 minutes after induction | *TP* |
| 27 | ccdB_K12_90_r1 | E.coli K12 with ccdB upregulation 90 minutes after induction | *TP* |
| 27 | ccdB_K12_120_r1 | E.coli K12 with ccdB upregulation 120 minutes after induction | *TP* |
| 28 | lacZ_K12_90_r1 | E.coli K12 with lacZ upregulation 90 minutes after induction | *TP* |
| 28 | lacZ_K12_120_r1 | E.coli K12 with lacZ upregulation 120 minutes after induction | *TP* |
| 29 | lacZ_MG1063_0_r1 | E.coli MG1063 (recA56 = recA-) with lacZ upregulation 0 minutes after induction | *TP* |
| 29 | lacZ_MG1063_30_r1 | E.coli MG1063 (recA56 = recA-) with lacZ upregulation 30 minutes after induction | *TP* |
| 29 | lacZ_MG1063_60_r1 | E.coli MG1063 (recA56 = recA-) with lacZ upregulation 60 minutes after induction | *TP* |
| 29 | lacZ_MG1063_90_r1 | E.coli MG1063 (recA56 = recA-) with lacZ upregulation 90 minutes after induction | *TP* |
| 29 | ccdB_MG1063_0_r1 | E.coli MG1063 (recA56 = recA-) with ccdB upregulation 0 minutes after induction | *TP* |
| 29 | ccdB_MG1063_30_r1 | E.coli MG1063 (recA56 = recA-) with ccdB upregulation 30 minutes after induction | *TP* |
| 29 | ccdB_MG1063_60_r1 | E.coli MG1063 (recA56 = recA-) with ccdB upregulation 60 minutes after induction | *TP* |
| 29 | lacZ_MG1063_0_r2 | E.coli MG1063 (recA56 = recA-) with lacZ upregulation 0 minutes after induction | *TP* |
| 29 | lacZ_MG1063_30_r2 | E.coli MG1063 (recA56 = recA-) with lacZ upregulation 30 minutes after induction | *TP* |
| 29 | lacZ_MG1063_60_r2 | E.coli MG1063 (recA56 = recA-) with lacZ upregulation 60 minutes after induction | *TP* |
| 29 | lacZ_MG1063_90_r2 | E.coli MG1063 (recA56 = recA-) with lacZ upregulation 90 minutes after induction | *TP* |
| 29 | lacZ_MG1063_120_r1 | E.coli MG1063 (recA56 = recA-) with lacZ upregulation 120 minutes after induction | *TP* |
| 29 | ccdB_MG1063_0_r2 | E.coli MG1063 (recA56 = recA-) with ccdB upregulation 0 minutes after induction | *TP* |
| 29 | ccdB_MG1063_30_r2 | E.coli MG1063 (recA56 = recA-) with ccdB upregulation 30 minutes after induction | *TP* |
| 29 | ccdB_MG1063_60_r2 | E.coli MG1063 (recA56 = recA-) with ccdB upregulation 60 minutes after induction | *TP* |
| 30 | ccdB_MG1063_90_r1 | E.coli MG1063 (recA56 = recA-) with ccdB upregulation 90 minutes after induction | *TP* |
| 30 | ccdB_MG1063_90_r2 | E.coli MG1063 (recA56 = recA-) with ccdB upregulation 90 minutes after induction | *TP* |
| 30 | ccdB_MG1063_120_r1 | E.coli MG1063 (recA56 = recA-) with ccdB upregulation 120 minutes after induction | *TP* |
| 31 | lacZ_W1863_0_r1 | E.coli W1863 wt lambda- with lacZ upregulation 0 minutes after induction | *TP* |
| 31 | ph5_r1 | strain K12 in LB pH adjusted to 5 with KOH and buffered with HOMOPIPES | [1] |
| 31 | ph5_r2 | strain K12 in LB pH adjusted to 5 with KOH and buffered with HOMOPIPES | [1] |
| 31 | ph5_r3 | strain K12 in LB pH adjusted to 5 with KOH and buffered with HOMOPIPES | [1] |
| 31 | ph5_r4 | strain K12 in LB pH adjusted to 5 with KOH and buffered with HOMOPIPES | [1] |
| 31 | ph5_r5 | strain K12 in LB pH adjusted to 5 with KOH and buffered with HOMOPIPES | [1] |
| 31 | ph7_r1 | strain K12 in LB pH adjusted to 7 with KOH and buffered with HOMOPIPES | [1] |
| 31 | ph7_r2 | strain K12 in LB pH adjusted to 7 with KOH and buffered with HOMOPIPES | [1] |
| 31 | ph7_r3 | strain K12 in LB pH adjusted to 7 with KOH and buffered with HOMOPIPES | [1] |
| 31 | ph7_r4 | strain K12 in LB pH adjusted to 7 with KOH and buffered with HOMOPIPES | [1] |
| 31 | ph7_r5 | strain K12 in LB pH adjusted to 7 with KOH and buffered with HOMOPIPES | [1] |
| 31 | ph8.7_r1 | strain K12 in LB pH adjusted to 8.7 with KOH and buffered with HOMOPIPES | [1] |
| 31 | ph8.7_r2 | strain K12 in LB pH adjusted to 8.7 with KOH and buffered with HOMOPIPES | [1] |
| 31 | ph8.7_r3 | strain K12 in LB pH adjusted to 8.7 with KOH and buffered with HOMOPIPES | [1] |
| 31 | ph8.7_r4 | strain K12 in LB pH adjusted to 8.7 with KOH and buffered with HOMOPIPES | [1] |
| 31 | ph8.7_r5 | strain K12 in LB pH adjusted to 8.7 with KOH and buffered with HOMOPIPES | [1] |
| 32 | lacZ_W1863_30_r1 | E.coli W1863 wt lambda- with lacZ upregulation 30 minutes after induction | *TP* |
| 32 | lacZ_W1863_60_r1 | E.coli W1863 wt lambda- with lacZ upregulation 60 minutes after induction | *TP* |
| 32 | ccdB_W1863_0_r1 | E.coli W1863 wt lambda- with ccdB upregulation 0 minutes after induction | *TP* |
| 32 | ccdB_W1863_30_r1 | E.coli W1863 wt lambda- with ccdB upregulation 30 minutes after induction | *TP* |
| 33 | lacZ_W1863_90_r1 | E.coli W1863 wt lambda- with lacZ upregulation 90 minutes after induction | *TP* |
| 33 | ccdB_W1863_60_r1 | E.coli W1863 wt lambda- with ccdB upregulation 60 minutes after induction | *TP* |
| 33 | ccdB_W1863_90_r1 | E.coli W1863 wt lambda- with ccdB upregulation 90 minutes after induction | *TP* |
| 34 | appY_KO9_r1 | aerobic growth of appY knock-out strain on M9 media with glucose | [2] |
| 34 | appY_KO9_r2 | aerobic growth of appY knock-out strain on M9 media with glucose | [2] |
| 34 | arcA_KO9_r1 | aerobic growth of arcA knock-out strain on M9 media with glucose | [2] |
| 34 | arcA_KO9_r2 | aerobic growth of arcA knock-out strain on M9 media with glucose | [2] |
| 34 | arcA_KO9_r3 | aerobic growth of arcA knock-out strain on M9 media with glucose | [2] |
| 34 | arcAfnr_KO9_r1 | aerobic growth of arcA/fnr double knock-out strain on M9 media with glucose | [2] |
| 34 | arcAfnr_KO9_r2 | aerobic growth of arcA/fnr double knock-out strain on M9 media with glucose | [2] |
| 34 | arcAfnr_KO9_r3 | aerobic growth of arcA/fnr double knock-out strain on M9 media with glucose | [2] |
| 34 | fnr_KO9_r1 | aerobic growth of fnr knock-out strain on M9 media with glucose | [2] |
| 34 | fnr_KO9_r2 | aerobic growth of fnr knock-out strain on M9 media with glucose | [2] |
| 34 | fnr_KO9_r3 | aerobic growth of fnr knock-out strain on M9 media with glucose | [2] |
| 34 | oxyR_KO9_r1 | aerobic growth of oxyR knock-out strain on M9 media with glucose | [2] |
| 34 | oxyR_KO9_r2 | aerobic growth of oxyR knock-out strain on M9 media with glucose | [2] |
| 34 | oxyR_KO9_r3 | aerobic growth of oxyR knock-out strain on M9 media with glucose | [2] |
| 34 | soxS_KO9_r1 | aerobic growth of soxS knock-out strain on M9 media with glucose | [2] |
| 34 | soxS_KO9_r2 | aerobic growth of soxS knock-out strain on M9 media with glucose | [2] |
| 34 | soxS_KO9_r3 | aerobic growth of soxS knock-out strain on M9 media with glucose | [2] |
| 34 | WT_O9_r1 | aerobic aerobic growth of wild-type strain on M9 media with glucose | [2] |
| 34 | WT_O9_r2 | aerobic aerobic growth of wild-type strain on M9 media with glucose | [2] |
| 34 | WT_O9_r3 | aerobic aerobic growth of wild-type strain on M9 media with glucose | [2] |
| 34 | arcA_KN9_r1 | anaerobic growth of arcA knock-out strain on M9 media with glucose | [2] |
| 34 | arcA_KN9_r2 | anaerobic growth of arcA knock-out strain on M9 media with glucose | [2] |
| 34 | arcA_KN9_r3 | anaerobic growth of arcA knock-out strain on M9 media with glucose | [2] |
| 34 | arcAfnr_KN9_r1 | anaerobic growth of arcA/fnr double knock-out strain on M9 media with glucose | [2] |
| 34 | arcAfnr_KN9_r2 | anaerobic growth of arcA/fnr double knock-out strain on M9 media with glucose | [2] |
| 34 | arcAfnr_KN9_r3 | anaerobic growth of arcA/fnr double knock-out strain on M9 media with glucose | [2] |
| 35 | appY_KO9_r3 | aerobic growth of appY knock-out strain on M9 media with glucose | [2] |
| 35 | fnr_KN9_r1 | anaerobic growth of fnr knock-out strain on M9 media with glucose | [2] |
| 35 | cybr_N_stat_r1 | BW30270 stationary phase, anaerobic on MOPS minimal media with glucose | [3] |
| 35 | cybr_N_stat_r2 | BW30270 stationary phase, anaerobic on MOPS minimal media with glucose | [3] |
| 36 | appY_KN9_r1 | anaerobic growth of appY knock-out strain on M9 media with glucose | [2] |
| 36 | appY_KN9_r2 | anaerobic growth of appY knock-out strain on M9 media with glucose | [2] |
| 36 | appY_KN9_r3 | anaerobic growth of appY knock-out strain on M9 media with glucose | [2] |
| 36 | fnr_KN9_r2 | anaerobic growth of fnr knock-out strain on M9 media with glucose | [2] |
| 36 | fnr_KN9_r3 | anaerobic growth of fnr knock-out strain on M9 media with glucose | [2] |
| 36 | oxyR_KN9_r1 | anaerobic growth of oxyR knock-out strain on M9 media with glucose | [2] |
| 36 | oxyR_KN9_r2 | anaerobic growth of oxyR knock-out strain on M9 media with glucose | [2] |
| 36 | oxyR_KN9_r3 | anaerobic growth of oxyR knock-out strain on M9 media with glucose | [2] |
| 36 | soxS_KN9_r1 | anaerobic growth of soxS knock-out strain on M9 media with glucose | [2] |
| 36 | soxS_KN9_r2 | anaerobic growth of soxS knock-out strain on M9 media with glucose | [2] |
| 36 | soxS_KN9_r3 | anaerobic growth of soxS knock-out strain on M9 media with glucose | [2] |
| 36 | WT_N9_r1 | anaerobic growth of wild-type strain on M9 media with glucose | [2] |
| 36 | WT_N9_r2 | anaerobic growth of wild-type strain on M9 media with glucose | [2] |
| 36 | WT_N9_r3 | anaerobic growth of wild-type strain on M9 media with glucose | [2] |
| 36 | WT_N9_r4 | anaerobic growth of wild-type strain on M9 media with glucose | [2] |
| 37 | yebF__U_N0075_r1 | yebF upregulation, 0.075 ug/ml norfloxacin | *TP* |
| 37 | yebF__U_N0075_r3 | yebF upregulation, 0.075 ug/ml norfloxacin | *TP* |
| 38 | yebF__U_N0075_r2 | yebF upregulation, 0.075 ug/ml norfloxacin | *TP* |
| 38 | dam___U_N0075_r2 | dam upregulation, 0.075 ug/ml norfloxacin | *TP* |
| 39 | WT_OPG_r1 | aerobic growth wild-type cells OD 0.2 on MOPS media with glucose | [4] |
| 39 | ast_pBADsup2_r1 | MG1655 OD 0.2 on MOPS media with arabinose controlled induction of an amber suppressor tRNA | [5] |
| 39 | ast_pBADsup2_r2 | MG1655 OD 0.2 on MOPS media with arabinose controlled induction of an amber suppressor tRNA | [5] |
| 39 | ast_pBADsup2_r3 | MG1655 OD 0.2 on MOPS media with arabinose controlled induction of an amber suppressor tRNA | [5] |
| 39 | ast_pBAD18_r1 | MG1655 OD 0.2 on MOPS media with empty pBAD18 vector | [5] |
| 39 | ast_pBAD18_r2 | MG1655 OD 0.2 on MOPS media with empty pBAD18 vector | [5] |
| 39 | ast_pBAD18_r3 | MG1655 OD 0.2 on MOPS media with empty pBAD18 vector | [5] |
| 39 | cybr_O_r1 | MG1655 log phase, aerobic on MOPS minimal media with glucose | [3] |
| 39 | cybr_O_r2 | MG1655 log phase, aerobic on MOPS minimal media with glucose | [3] |
| 39 | cybr_O_log_r1 | BW30270 log phase, aerobic on MOPS minimal media with glucose | [3] |
| 39 | cybr_O_log_r2 | BW30270 log phase, aerobic on MOPS minimal media with glucose | [3] |
| 40 | luc___U_N0075_r1 | luc upregulation, 0.075 ug/ml norfloxacin | *TP* |
| 40 | luc___U_N0075_r3 | luc upregulation, 0.075 ug/ml norfloxacin | *TP* |
| 41 | luc___U_N0075_r2 | luc upregulation, 0.075 ug/ml norfloxacin | *TP* |
| 41 | zipA__U_N0075_r2 | zipA upregulation, 0.075 ug/ml norfloxacin | *TP* |
| 41 | zipA__U_N0075_r3 | zipA upregulation, 0.075 ug/ml norfloxacin | *TP* |
| 41 | cspF__U_N0075_r1 | cspF upregulation, 0.075 ug/ml norfloxacin | *TP* |
| 41 | cspF__U_N0075_r2 | cspF upregulation, 0.075 ug/ml norfloxacin | *TP* |
| 41 | dam___U_N0075_r1 | dam upregulation, 0.075 ug/ml norfloxacin | *TP* |
| 41 | dam___U_N0075_r3 | dam upregulation, 0.075 ug/ml norfloxacin | *TP* |
| 41 | fis___U_N0075_r2 | fis upregulation, 0.075 ug/ml norfloxacin | *TP* |
| 41 | gcvR__U_N0075_r2 | gcvR upregulation, 0.075 ug/ml norfloxacin | *TP* |
| 41 | nrdA__U_N0075_r2 | nrdA upregulation, 0.075 ug/ml norfloxacin | *TP* |
| 41 | ruvC__U_N0075_r2 | ruvC upregulation, 0.075 ug/ml norfloxacin | *TP* |
| 42 | WT_OPG_r2 | aerobic growth wild-type cells OD 0.2 on MOPS media with glucose | [4] |
| 42 | WT_OPG_r3 | aerobic growth wild-type cells OD 0.2 on MOPS media with glucose | [4] |
| 42 | WT_OPG_r4 | aerobic growth wild-type cells OD 0.2 on MOPS media with glucose | [4] |
| 42 | WT_OPG_r5 | aerobic growth wild-type cells OD 0.2 on MOPS media with glucose | [4] |
| 42 | WT_OPG1_r1 | aerobic growth wild-type late log phase 90min MOPS media with glucose | [4] |
| 42 | WT_OPG1_r2 | aerobic growth wild-type late log phase 90 min MOPS media with glucose | [4] |
| 42 | WT_OPG1_r3 | aerobic growth wild-type late log phase 90 min MOPS media with glucose | [4] |
| 42 | WT_OPGA_r1 | aerobic growth wild-type log phase MOPS media with glucose 10 min acid shock pH 2 | [4] |
| 42 | WT_OPGA_r2 | aerobic growth wild-type log phase MOPS media with glucose 10 min acid shock pH 2 | [4] |
| 42 | WT_OPGC1_r1 | aerobic growth wild-type log phase MOPS media with glucose 10 min ciprofloxacin 20 ng/ml | [4] |
| 42 | WT_OPGC2_r1 | aerobic growth wild-type log phase MOPS media with glucose 30 min ciprofloxacin 20 ng/ml | [4] |
| 42 | cspA_KOPG_r1 | cspA::Tn5 mutant aerobic growth wild-type log phase MOPS media | [4] |
| 42 | dps_KOPG_r1 | dps::Tn5 mutant aerobic growth wild-type log phase MOPS media | [4] |
| 42 | dps_KOPG_r2 | dps::Tn5 mutant aerobic growth wild-type log phase MOPS media | [4] |
| 42 | dps_KOPG_r3 | dps::Tn5 mutant aerobic growth wild-type log phase MOPS media | [4] |
| 42 | hupB_KOPG_r1 | hupB::Tn5 mutant aerobic growth wild-type log phase MOPS media | [4] |
| 42 | fnr_DfnrAerobic_r1 | MG1655 with fnr deletion OD 0.2 grown aerobically | [6] |
| 42 | fnr_DfnrAerobic_r2 | MG1655 with fnr deletion OD 0.2 grown aerobically | [6] |
| 42 | fnr_DfnrAerobic_r3 | MG1655 with fnr deletion OD 0.2 grown aerobically | [6] |
| 43 | zipA__U_N0075_r1 | zipA upregulation, 0.075 ug/ml norfloxacin | *TP* |
| 44 | WT_OPA_r1 | aerobic growth wild-type cells OD 0.2 on MOPS media with acetate | [4] |
| 44 | WT_OPA_r2 | aerobic growth wild-type cells OD 0.2 on MOPS media with acetate | [4] |
| 44 | WT_OPY_r1 | aerobic growth wild-type cells OD 0.2 on MOPS media with glycerol | [4] |
| 44 | WT_OPY_r2 | aerobic growth wild-type cells OD 0.2 on MOPS media with glycerol | [4] |
| 44 | WT_OPL_r1 | aerobic growth wild-type cells OD 0.2 on MOPS media with proline | [4] |
| 44 | WT_OPL_r2 | aerobic growth wild-type cells OD 0.2 on MOPS media with proline | [4] |
| 44 | WT_OPG2_r1 | aerobic growth wild-type stationary phase 135 min MOPS media with glucose | [4] |
| 44 | WT_OPG2_r2 | aerobic growth wild-type stationary phase 135 min MOPS media with glucose | [4] |
| 44 | csf_succinate_r1 | aerobic growth wild-type cells OD 0.2 on MOPS media with acetate | [7] |
| 44 | csf_succinate_r2 | aerobic growth wild-type cells OD 0.2 on MOPS media with acetate | [7] |
| 45 | WT_OPG3_r1 | aerobic growth wild-type stationary phase 330 min MOPS media with glucose | [4] |
| 45 | WT_OPG3_r2 | aerobic growth wild-type stationary phase 330 min MOPS media with glucose | [4] |
| 45 | WT_OPG4_r1 | aerobic growth wild-type stationary phase 480 min MOPS media with glucose | [4] |
| 45 | WT_OPG4_r2 | aerobic growth wild-type stationary phase 480 min MOPS media with glucose | [4] |
| 45 | WT_OPG5_r1 | aerobic growth wild-type stationary phase 720 min MOPS media with glucose | [4] |
| 45 | WT_OPG5_r2 | aerobic growth wild-type stationary phase 720 min MOPS media with glucose | [4] |
| 45 | dps_KOPG2_r1 | dps::Tn5 mutant aerobic growth wild-type stationary phase 240 min MOPS media | [4] |
| 45 | dps_KOPG2_r2 | dps::Tn5 mutant aerobic growth wild-type stationary phase 240 min MOPS media | [4] |
| 45 | dps_KOPG3_r1 | dps::Tn5 mutant aerobic growth wild-type stationary phase 480 min MOPS media | [4] |
| 45 | cybr_O_stat_r1 | BW30270 stationary phase, aerobic on MOPS minimal media with glucose | [3] |
| 45 | cybr_O_stat_r2 | BW30270 stationary phase, aerobic on MOPS minimal media with glucose | [3] |
| 46 | WT_OPGH_r1 | aerobic growth wild-type log phase MOPS media with glucose 10min heat shock 50°C | [4] |
| 46 | crp_KOPG_r1 | crp::Tn5 mutant aerobic growth wild-type log phase MOPS media | [4] |
| 46 | crp_KOPG_r2 | crp::Tn5 mutant aerobic growth wild-type log phase MOPS media | [4] |
| 46 | crp_KOPG_r3 | crp::Tn5 mutant aerobic growth wild-type log phase MOPS media | [4] |
| 46 | hns_KOPG_r1 | hns::Tn5 mutant aerobic growth wild-type log phase MOPS media | [4] |
| 46 | hns_KOPG_r2 | hns::Tn5 mutant aerobic growth wild-type log phase MOPS media | [4] |
| 46 | hns_KOPG_r3 | hns::Tn5 mutant aerobic growth wild-type log phase MOPS media | [4] |
| 47 | MGD1_t0_r1 | strain BL21 (DE3) with mussel defensin protein MGD1 on T7 controllable plasmid preinduction | [8] |
| 47 | MGD1_t30_r2 | strain BL21 (DE3) with mussel defensin protein MGD1 on T7 controllable plasmid 30min postinduction with 1mM IPTG | [8] |
| 47 | pET3d_t0_r2 | strain BL21 (DE3) with pET3d plasmid and no insert dna (makes 26 amino acid polypeptide) preinduction | [8] |
| 48 | MGD1_t0_r2 | strain BL21 (DE3) with mussel defensin protein MGD1 on T7 controllable plasmid preinduction | [8] |
| 48 | pET3d_t0_r1 | strain BL21 (DE3) with pET3d plasmid and no insert dna (makes 26 amino acid polypeptide) preinduction | [8] |
| 48 | pepAA_t0_r1 | strain BL21 (DE3) with T7 controllable synthetic peptide containing least abundant E. coli amino acids preinduction | [8] |
| 48 | pepAA_t0_r2 | strain BL21 (DE3) with T7 controllable synthetic peptide containing least abundant E. coli amino acids preinduction | [8] |
| 48 | pepCO_t0_r1 | strain BL21 (DE3) with T7 controllable synthetic peptide containing most abundant E. coli amino acids preinduction | [8] |
| 48 | pepCO_t0_r2 | strain BL21 (DE3) with T7 controllable synthetic peptide containing most abundant E. coli amino acids preinduction | [8] |
| 48 | pepCO_t30_r1 | strain BL21 (DE3) with T7 controllable synthetic peptide containing most abundant E. coli amino acids 30 min postinduction with 1mM IPTG | [8] |
| 49 | b2618_U_N0075_r1 | b2618 upregulation, 0.075 ug/ml norfloxacin | *TP* |
| 49 | b2618_U_N0075_r3 | b2618 upregulation, 0.075 ug/ml norfloxacin | *TP* |
| 49 | bcp___U_N0075_r1 | bcp upregulation, 0.075 ug/ml norfloxacin | *TP* |
| 49 | bcp___U_N0075_r3 | bcp upregulation, 0.075 ug/ml norfloxacin | *TP* |
| 49 | cpxR__U_N0075_r1 | cpxR upregulation, 0.075 ug/ml norfloxacin | *TP* |
| 49 | cpxR__U_N0075_r3 | cpxR upregulation, 0.075 ug/ml norfloxacin | *TP* |
| 49 | crcB__U_N0075_r1 | crcB upregulation, 0.075 ug/ml norfloxacin | *TP* |
| 49 | crcB__U_N0075_r3 | crcB upregulation, 0.075 ug/ml norfloxacin | *TP* |
| 49 | crp___U_N0075_r3 | crp upregulation, 0.075 ug/ml norfloxacin | *TP* |
| 49 | cspF__U_N0075_r3 | cspF upregulation, 0.075 ug/ml norfloxacin | *TP* |
| 49 | dnaA__U_N0075_r3 | dnaA upregulation, 0.075 ug/ml norfloxacin | *TP* |
| 49 | dnaN__U_N0075_r3 | dnaN upregulation, 0.075 ug/ml norfloxacin | *TP* |
| 49 | dnaT__U_N0075_r3 | dnaT upregulation, 0.075 ug/ml norfloxacin | *TP* |
| 49 | era___U_N0075_r2 | era upregulation, 0.075 ug/ml norfloxacin | *TP* |
| 49 | era___U_N0075_r3 | era upregulation, 0.075 ug/ml norfloxacin | *TP* |
| 49 | fis___U_N0075_r1 | fis upregulation, 0.075 ug/ml norfloxacin | *TP* |
| 49 | fis___U_N0075_r3 | fis upregulation, 0.075 ug/ml norfloxacin | *TP* |
| 49 | fklB__U_N0075_r1 | fklB upregulation, 0.075 ug/ml norfloxacin | *TP* |
| 49 | fklB__U_N0075_r2 | fklB upregulation, 0.075 ug/ml norfloxacin | *TP* |
| 49 | fklB__U_N0075_r3 | fklB upregulation, 0.075 ug/ml norfloxacin | *TP* |
| 49 | folA__U_N0075_r1 | folA upregulation, 0.075 ug/ml norfloxacin | *TP* |
| 49 | folA__U_N0075_r3 | folA upregulation, 0.075 ug/ml norfloxacin | *TP* |
| 49 | galF__U_N0075_r3 | galF upregulation, 0.075 ug/ml norfloxacin | *TP* |
| 49 | gcvR__U_N0075_r3 | gcvR upregulation, 0.075 ug/mL norfloxacin | *TP* |
| 49 | gyrA__U_N0075_r3 | gyrA upregulation, 0.075 ug/mL norfloxacin | *TP* |
| 49 | hlpA__U_N0075_r3 | hlpA upregulation, 0.075 ug/mL norfloxacin | *TP* |
| 49 | ldrA__U_N0075_r3 | ldrA upregulation, 0.075 ug/mL norfloxacin | *TP* |
| 49 | mcrB__U_N0075_r1 | mcrB upregulation, 0.075 ug/mL norfloxacin | *TP* |
| 49 | mcrB__U_N0075_r2 | mcrB upregulation, 0.075 ug/mL norfloxacin | *TP* |
| 49 | mcrB__U_N0075_r3 | mcrB upregulation, 0.075 ug/mL norfloxacin | *TP* |
| 49 | mcrC__U_N0075_r1 | mcrC upregulation, 0.075 ug/mL norfloxacin | *TP* |
| 49 | mcrC__U_N0075_r2 | mcrC upregulation, 0.075 ug/mL norfloxacin | *TP* |
| 49 | mcrC__U_N0075_r3 | mcrC upregulation, 0.075 ug/mL norfloxacin | *TP* |
| 49 | menB__U_N0075_r3 | menB upregulation, 0.075 ug/mL norfloxacin | *TP* |
| 49 | minE__U_N0075_r3 | minE upregulation, 0.075 ug/mL norfloxacin | *TP* |
| 49 | pyrC__U_N0075_r1 | pyrC upregulation, 0.075 ug/mL norfloxacin | *TP* |
| 49 | pyrC__U_N0075_r2 | pyrC upregulation, 0.075 ug/mL norfloxacin | *TP* |
| 49 | pyrC__U_N0075_r3 | pyrC upregulation, 0.075 ug/mL norfloxacin | *TP* |
| 49 | rimI__U_N0075_r2 | rimI upregulation, 0.075 ug/mL norfloxacin | *TP* |
| 49 | uspA__U_N0075_r3 | uspA upregulation, 0.075 ug/mL norfloxacin | *TP* |
| 50 | b2618_U_N0075_r2 | b2618 upregulation, 0.075 ug/ml norfloxacin | *TP* |
| 50 | crcB__U_N0075_r2 | crcB upregulation, 0.075 ug/ml norfloxacin | *TP* |
| 50 | crp___U_N0075_r1 | crp upregulation, 0.075 ug/ml norfloxacin | *TP* |
| 50 | era___U_N0075_r1 | era upregulation, 0.075 ug/ml norfloxacin | *TP* |
| 50 | gyrA__U_N0075_r2 | gyrA upregulation, 0.075 ug/mL norfloxacin | *TP* |
| 50 | ldrA__U_N0075_r1 | ldrA upregulation, 0.075 ug/mL norfloxacin | *TP* |
| 50 | ldrA__U_N0075_r2 | ldrA upregulation, 0.075 ug/mL norfloxacin | *TP* |
| 50 | nupC__U_N0075_r1 | nupC upregulation, 0.075 ug/mL norfloxacin | *TP* |
| 50 | rstB__U_N0075_r2 | rstB upregulation, 0.075 ug/mL norfloxacin | *TP* |
| 51 | MGD1_t30_r1 | strain BL21 (DE3) with mussel defensin protein MGD1 on T7 controllable plasmid 30 min postinduction with 1mM IPTG | [8] |
| 52 | pET3d_t30_r1 | strain BL21 (DE3) with pET3d plasmid and no insert dna (makes 26 amino acid polypeptide) 30 min postinduction with 1 mM IPTG | [8] |
| 52 | pET3d_t30_r2 | strain BL21 (DE3) with pET3d plasmid and no insert dna (makes 26 amino acid polypeptide) 30 min postinduction with 1 mM IPTG | [8] |
| 52 | pepAA_t30_r1 | strain BL21 (DE3) with T7 controllable synthetic peptide containing least abundant E. coli amino acids 30 min postinduction with 1 mM IPTG | [8] |
| 52 | pepAA_t30_r2 | strain BL21 (DE3) with T7 controllable synthetic peptide containing least abundant E. coli amino acids 30 min postinduction with 1 mM IPTG | [8] |
| 52 | pepCO_t30_r2 | strain BL21 (DE3) with T7 controllable synthetic peptide containing most abundant E. coli amino acids 30 min postinduction with 1 mM IPTG | [8] |
| 53 | bcp___U_N0075_r2 | bcp upregulation, 0.075 ug/ml norfloxacin | *TP* |
| 53 | cpxR__U_N0075_r2 | cpxR upregulation, 0.075 ug/ml norfloxacin | *TP* |
| 53 | crp___U_N0075_r2 | crp upregulation, 0.075 ug/ml norfloxacin | *TP* |
| 53 | menB__U_N0075_r2 | menB upregulation, 0.075 ug/mL norfloxacin | *TP* |
| 53 | uspA__U_N0075_r2 | uspA upregulation, 0.075 ug/mL norfloxacin | *TP* |
| 54 | fnr_wtAnaerobic_r1 | MG1655 OD 0.1 grown anaerobically | [6] |
| 54 | fnr_wtAnaerobic_r2 | MG1655 OD 0.1 grown anaerobically | [6] |
| 54 | fnr_wtAnaerobic_r3 | MG1655 OD 0.1 grown anaerobically | [6] |
| 54 | fnr_DfnrAnaerobic_r1 | MG1655 with fnr deletion OD 0.1 grown anaerobically | [6] |
| 54 | fnr_DfnrAnaerobic_r2 | MG1655 with fnr deletion OD 0.1 grown anaerobically | [6] |
| 54 | fnr_DfnrAnaerobic_r3 | MG1655 with fnr deletion OD 0.1 grown anaerobically | [6] |
| 54 | fnr_DfnrAnaerobic_r4 | MG1655 with fnr deletion OD 0.1 grown anaerobically | [6] |
| 54 | cybr_N_log_r1 | BW30270 log phase, anaerobic on MOPS minimal media with glucose | [3] |
| 54 | cybr_N_log_r2 | BW30270 log phase, anaerobic on MOPS minimal media with glucose | [3] |
| 55 | har_S0_R_noIPTG_r1 | MG1655 with pPROEx-CAT plasmid late log phase in LB with glucose and MgSO_4_ | [9] |
| 55 | har_S0_R_noIPTG_r4 | MG1655 with pPROEx-CAT plasmid late log phase in LB with glucose and MgSO_4_ | [9] |
| 55 | har_S0_R_noIPTG_r2 | MG1655 with pPROEx-CAT plasmid late log phase in LB with glucose and MgSO_4_ | [9] |
| 55 | har_S0_R_noIPTG_r5 | MG1655 with pPROEx-CAT plasmid late log phase in LB with glucose and MgSO_4_ | [9] |
| 55 | har_S0_R_noIPTG_r3 | MG1655 with pPROEx-CAT plasmid late log phase in LB with glucose and MgSO_4_ | [9] |
| 55 | har_S1_R_noIPTG_r1 | MG1655 with pPROEx-CAT plasmid late log phase +1hr in LB with glucose and MgSO4 | [9] |
| 55 | har_S1_R_noIPTG_r2 | MG1655 with pPROEx-CAT plasmid late log phase +1hr in LB with glucose and MgSO4 | [9] |
| 55 | har_S1_R_noIPTG_r3 | MG1655 with pPROEx-CAT plasmid late log phase +1hr in LB with glucose and MgSO4 | [9] |
| 55 | har_S0_noIPTG_r1 | MG1655 late log phase in LB with glucose and MgSO_4_ | [9] |
| 55 | har_S0_noIPTG_r2 | MG1655 late log phase in LB with glucose and MgSO_4_ | [9] |
| 55 | har_S0_noIPTG_r3 | MG1655 late log phase in LB with glucose and MgSO_4_ | [9] |
| 55 | har_S1_noIPTG_r1 | MG1655 late log phase +1hr in LB with glucose and MgSO_4_ | [9] |
| 55 | har_S1_noIPTG_r2 | MG1655 late log phase +1hr in LB with glucose and MgSO_4_ | [9] |
| 55 | har_S1_noIPTG_r3 | MG1655 late log phase +1hr in LB with glucose and MgSO_4_ | [9] |
| 55 | har_S4_noIPTG_r1 | MG1655 late log phase +4hr in LB with glucose and MgSO_4_ | [9] |
| 55 | har_S4_noIPTG_r2 | MG1655 late log phase +4hr in LB with glucose and MgSO_4_ | [9] |
| 55 | har_S4_noIPTG_r3 | MG1655 late log phase +4hr in LB with glucose and MgSO_4_ | [9] |
| 55 | har_S1_IPTG_r1 | MG1655 late log phase +1hr in LB with glucose and MgSO_4_ and IPTG | [9] |
| 55 | har_S1_IPTG_r2 | MG1655 late log phase +1hr in LB with glucose and MgSO_4_ and IPTG | [9] |
| 55 | har_S1_IPTG_r3 | MG1655 late log phase +1hr in LB with glucose and MgSO_4_ and IPTG | [9] |
| 55 | har_S4_IPTG_r1 | MG1655 late log phase +4hr in LB with glucose and MgSO_4_ and IPTG | [9] |
| 55 | har_S4_IPTG_r2 | MG1655 late log phase +4hr in LB with glucose and MgSO_4_ and IPTG | [9] |
| 55 | har_S4_IPTG_r3 | MG1655 late log phase +4hr in LB with glucose and MgSO_4_ and IPTG | [9] |
| 56 | har_S4_R_noIPTG_r1 | MG1655 with pPROEx-CAT plasmid late log phase +4hr in LB with glucose and MgSO4 | [9] |
| 56 | har_S4_R_noIPTG_r2 | MG1655 with pPROEx-CAT plasmid late log phase +4hr in LB with glucose and MgSO4 | [9] |
| 56 | har_S4_R_noIPTG_r3 | MG1655 with pPROEx-CAT plasmid late log phase +4hr in LB with glucose and MgSO4 | [9] |
| 56 | har_S1_R_IPTG_r1 | MG1655 with pPROEx-CAT plasmid late log phase +1hr in LB with glucose and MgSO4 and IPTG | [9] |
| 56 | har_S1_R_IPTG_r2 | MG1655 with pPROEx-CAT plasmid late log phase +1hr in LB with glucose and MgSO4 and IPTG | [9] |
| 56 | har_S1_R_IPTG_r3 | MG1655 with pPROEx-CAT plasmid late log phase +1hr in LB with glucose and MgSO4 and IPTG | [9] |
| 56 | har_S4_R_IPTG_r1 | MG1655 with pPROEx-CAT plasmid late log phase +4hr in LB with glucose and MgSO4 and IPTG | [9] |
| 56 | har_S4_R_IPTG_r2 | MG1655 with pPROEx-CAT plasmid late log phase +4hr in LB with glucose and MgSO4 and IPTG | [9] |
| 56 | har_S4_R_IPTG_r3 | MG1655 with pPROEx-CAT plasmid late log phase +4hr in LB with glucose and MgSO4 and IPTG | [9] |
| 57 | cybr_KNO_N_r1 | MG1655 log phase, anaerobic with nitrate on MOPS minimal media with glucose | [3] |
| 57 | cybr_KNO_N_r2 | MG1655 log phase, anaerobic with nitrate on MOPS minimal media with glucose | [3] |
| 57 | cybr_N_r1 | MG1655 log phase, anaerobic on MOPS minimal media with glucose | [3] |
| 57 | cybr_N_r2 | MG1655 log phase, anaerobic on MOPS minimal media with glucose | [3] |
| 58 | ik_L2_T2.5_r1 | K12 EMG2 on LB with 0.2 percent glucose, 2.5 hours post-incubation | *TP* |
| 58 | ik_L2_T3_r1 | K12 EMG2 on LB with 0.2 percent glucose, 3 hours post-incubation | *TP* |
| 58 | ik_L2_T3.5_r1 | K12 EMG2 on LB with 0.2 percent glucose, 3.5 hours post-incubation | *TP* |
| 58 | ik_L2_T4_r1 | K12 EMG2 on LB with 0.2 percent glucose, 4 hours post-incubation | *TP* |
| 58 | ik_L2_T4.5_r1 | K12 EMG2 on LB with 0.2 percent glucose, 4.5 hours post-incubation | *TP* |
| 58 | ik_H2_T2.5_r1 | K12 EMG2 on LB with 0.4 percent glucose, 2.5 hours post-incubation | *TP* |
| 58 | ik_H2_T3_r1 | K12 EMG2 on LB with 0.4 percent glucose, 3 hours post-incubation | *TP* |
| 58 | ik_H2_T3.5_r1 | K12 EMG2 on LB with 0.4 percent glucose, 3.5 hours post-incubation | *TP* |
| 59 | ik_L2_T5_r1 | K12 EMG2 on LB with 0.2 percent glucose, 5 hours post-incubation | *TP* |
| 59 | ik_L2_T5.5_r1 | K12 EMG2 on LB with 0.2 percent glucose, 5.5 hours post-incubation | *TP* |
| 59 | ik_L2_T6_r1 | K12 EMG2 on LB with 0.2 percent glucose, 6 hours post-incubation | *TP* |
| 59 | ik_H2_T4_r1 | K12 EMG2 on LB with 0.4 percent glucose, 4 hours post-incubation | *TP* |
| 59 | ik_H2_T4.5_r1 | K12 EMG2 on LB with 0.4 percent glucose, 4.5 hours post-incubation | *TP* |
| 59 | ik_H2_T5_r1 | K12 EMG2 on LB with 0.4 percent glucose, 5 hours post-incubation | *TP* |
| 59 | ik_H2_T5.5_r1 | K12 EMG2 on LB with 0.4 percent glucose, 5.5 hours post-incubation | *TP* |
| 59 | ik_H2_T6_r1 | K12 EMG2 on LB with 0.4 percent glucose, 6 hours post-incubation | *TP* |
| 59 | ik_H2_T8_r1 | K12 EMG2 on LB with 0.4 percent glucose, 8 hours post-incubation | *TP* |
| 60 | ik_L2_T8_r1 | K12 EMG2 on LB with 0.2 percent glucose, 8 hours post-incubation | *TP* |

Experiments were clustered with the complete-linkage algorithm using inverse CLR scores computed for the microarrays (not the genes) as the pairwise distance metric. The tree was pruned at 60 clusters.

**Table S4 References**

1. Maurer LM, Yohannes E, Bondurant SS, Radmacher M, Slonczewski JL (2005) pH regulates genes for flagellar motility, catabolism, and oxidative stress in Escherichia coli K-12. J Bacteriol 187: 304-319.

2. Covert MW, Knight EM, Reed JL, Herrgard MJ, Palsson BO (2004) Integrating high-throughput and computational data elucidates bacterial networks. Nature 429: 92-96.

3. Brokx SJ, Ellison M, Locke T, Bottorff D, Frost L, et al. (2004) Genome-wide analysis of lipoprotein expression in Escherichia coli MG1655. J Bacteriol 186: 3254-3258.

4. Allen TE, Herrgard MJ, Liu M, Qiu Y, Glasner JD, et al. (2003) Genome-scale analysis of the uses of the Escherichia coli genome: model-driven analysis of heterogeneous data sets. J Bacteriol 185: 6392-6399.

5. Herring CD, Blattner FR (2004) Global transcriptional effects of a suppressor tRNA and the inactivation of the regulator frmR. J Bacteriol 186: 6714-6720.

6. Kang Y, Weber KD, Qiu Y, Kiley PJ, Blattner FR (2005) Genome-wide expression analysis indicates that FNR of Escherichia coli K-12 regulates a large number of genes of unknown function. J Bacteriol 187: 1135-1160.

7. Liu M, Durfee T, Cabrera JE, Zhao K, Jin DJ, et al. (2005) Global transcriptional programs reveal a carbon source foraging strategy by Escherichia coli. J Biol Chem 280: 15921-15927.

8. Bonomo J, Gill RT (2005) Amino acid content of recombinant proteins influences the metabolic burden response. Biotechnol Bioeng 90: 116-126.

9. Haddadin FT, Harcum SW (2005) Transcriptome profiles for high-cell-density recombinant and wild-type Escherichia coli. Biotechnol Bioeng 90: 127-153.
